# Adaptation of Mycobacteria to Growth Conditions: A Theoretical Analysis of Changes in Gene Expression Revealed by Microarrays

**DOI:** 10.1371/journal.pone.0059883

**Published:** 2013-04-12

**Authors:** Robert Ashley Cox, Maria Jesus Garcia

**Affiliations:** 1 Division of Mycobacterial Research, National Institute for Medical Research, London, United Kingdom; 2 Departamento de Medicina Preventiva, Facultad de Medicina, Universidad Autonoma de Madrid, Spain; University College Dublin, Ireland

## Abstract

**Background:**

Microarray analysis is a powerful technique for investigating changes in gene expression. Currently, results (*r*-values) are interpreted empirically as either unchanged or up- or down-regulated. We now present a mathematical framework, which relates *r*-values to the macromolecular properties of population-average cells. The theory is illustrated by the analysis of published data for two species; namely, *Mycobacterium bovis* BCG Pasteur and *Mycobacterium smegmatis* mc^2^ 155. Each species was grown in a chemostat at two different growth rates. Application of the theory reveals the growth rate dependent changes in the mycobacterial proteomes.

**Principal Findings:**

The *r*-value *r*_(i)_ of any ORF (ORF_(i)_) encoding protein *p*_(i)_ was shown to be equal to the ratio of the concentrations of *p*_(i)_ and so directly proportional to the ratio of the numbers of copies of *p*_(i)_ per population-average cells of the two cultures. The proportionality constant can be obtained from the ratios DNA: RNA: protein. Several subgroups of ORFs were identified because they shared a particular *r-*value. Histograms of the number of ORFs versus the expression ratio were simulated by combining the particular *r*-values of several subgroups of ORFs. The largest subgroup was ORF_(j)_ (*r*_(j)_  = 1.00± SD) which was estimated to comprise respectively 59% and 49% of ORFs of *M. bovis* BCG Pasteur and *M. smegmatis* mc^2^ 155. The standard deviations reflect the properties of the cDNA preparations investigated.

**Significance:**

The analysis provided a quantitative view of growth rate dependent changes in the proteomes of the mycobacteria studied. The majority of the ORFs were found to be constitutively expressed. In contrast, the protein compositions of the outer permeability barriers and cytoplasmic membranes were found to be dependent on growth rate; thus illustrating the response of bacteria to their environment. The theoretical approach applies to any cultivatable bacterium under a wide range of growth conditions.

## Introduction

‘*Trying to make sense of the wealth of data produced by microarray experiments is immensely exciting but overwhelming*’ [1].

The complete genome sequence of the model organism *Escherichia coli* K12 was reported in 1997 [2]; hundreds of complete bacterial genome sequences are now available. The acquisition of genomic sequence data has stimulated the search for methods for studying transcription patterns of the entire genome. Microarray analysis was an early invention (for review, see [3]). This method is based on the competitive hybridization of cDNA copies of experimental and reference samples of cellular RNA to immobilized DNA. The results are expressed as a set of ratios (*r*-values) represented by *r*_(i)_ *et cetera* where the subscript “i”refers to an open reading frame ORF_(i)_.

The clinical importance of the pathogens *Mycobacterium tuberculosis* and *Mycobacterium leprae* stimulated both the elucidation of their genomic sequences [4], [5] and studies of gene expression by microarray analysis. Such studies have increased our knowledge of both the bacterial and pathogenic properties of *M. tuberculosis* and members of the *M. tuberculosis* complex [6]–[10].

Compared with other genera, mycobacteria are characterized by their slow or very slow growth. Traditionally, the two groups are termed fast- and slow-growing mycobacteria. Two microarray studies of mycobacteria grown in chemostats at different growth rates were available, each corresponding to one or other of the above-mentioned groups. The ‘slow-grower’ *Mycobacterium bovis* BCG (strain Pasteur ATCC 35748) (BCG-Pasteur) was grown at a slow rate and at the near to the maximum rate (a threefold change) and the two patterns of transcription were compared [11]. The transcription patterns of the fast grower *Mycobacterium smegmatis* mc^2^ 155 (Msmeg) grown at slow and fast rates (a fifteen fold change) were also reported [12]. A control was provided by wild type *M.tuberculosis* and a dosR minus mutant that were shown to grow at the same rate and which were compared by microarray analysis [1]. These three reports provide the platform for this study. Our aim was first to identify the similarities and differences between the two sets of data and then to explain them by using a mathematical framework. The benefits of this approach are that *r*-values may be expressed in terms of properties of population-average cells [13] and that significance is given to *r*-values in the range 0.51–1.99 as well as to the few ORFs which are ‘up-regulated’ twofold or more (*r*>2.0) or ‘down regulated’ twofold or more (*r*<0.5).

We define the expression of ORF_(i),_ as the synthesis of a copy of the encoded protein *p*_(i)_. Gene expression comprises two stages; namely, transcription and translation. In bacteria the two stages are coupled [14] so that ribosomes translate codons of ORF_(i)_ as fast as they are synthesized; that is, the rate of peptide chain elongation *ε*_aa(i)_ is equal to the rate of codon synthesis.

This definition of gene expression can be formulated mathematically, as shown by the following three equations which are further explained in the Methods section (see equations D1–D6). Symbols used are listed in Table 1.

**Table 1:** Definitions of Variables.

Symbol	Definition of variable (units in parentheses)
*m*_dc(av)_	Dry cell mass (femtograms) per population-average cell.
*m*_RNA(av)_	Mass (femtograms) of RNA per population-average cell.
*n*_aa(av)_	Number of amino acid residues of the protein fraction of population-average cells.
*n*′_cells,_ *n*′′_cells_	Numbers of population-average cells needed for synthesis of 1 pg of cDNA probe.
*n*_c–p(i)_, *n*_c–p(j)_, *n*_c–p(k)_	Gross number of copies per population-average cell of proteins *p*_(i},_ *p*_(j}_ and *p*_(k)_ encoded by ORF_(i),_ ORF_(j)_ and ORF_(k)_ respectively.
*n* ^#^_c–p(i)_, *n* ^#^_c–p(j)_, *n* ^#^_c–p(k)_	Apparent number, per population-average cell, of a reference culture of copies of, respectively, proteins *p*_(i},_ *p*_(j}_ and *p*_(k)._
n*_c–p(i)_, n*_c–p(j)_, n*_c–p(k)_	Apparent number, per population-average cell, of an experimental culture of copies of, respectively, proteins *p_(_*_i},_ *p*_(j}_ and *p*_(k)._
*n*_R(av)_	The number of ribosomes per population-average cell.
*n*_tr(i)_, *n*_tr(j)_, *n*_tr(k)_	Numbers, per population-average cell, of transcripts of ORF_(i)_, ORF_(j)_ and ORF_(k)_ respectively.
ORF_(i)_	Open reading frame encoding protein *p*_(i}_, represents any ORF.
ORF_(j)_	ORF_(j)_ encoding protein *p*_(j)_, represents a subgroup of ORF_(i)_ for which *r*_(j)_ = 1 independent of growth rate. Expression of these proteins is considered constitutive.
ORF_(k)_	ORF_(k)_ encoding protein *p*_(k)_, represents a subgroup of ORF_(i)_ that encode proteins whose abundance is directly proportional to *n*R_(av_).
*f* ^#^, *f* *	Fluorescence of cDNA samples prepared from reference and experimental cells respectively
*r*	The *r*-value (*f* */*f* ^#^).
*r*_(i)_, *r*_(j)_, *r*_(k)_	Expression ratio for ORF_(i)_, ORF_(j)_ and ORF_(k)_ respectively.
*β*_R_	Fraction of ribosomes, per population-average cell, that is actively synthesizing protein.
*ε*_aa(av)_	The polypeptide chain elongation rate (amino acids h^−1^) of the protein fraction of population-average cells.
*ε*_aa(i)_, *ε*_aa(j)_, *ε*_aa(k)_	The polypeptide chain elongation rates of proteins *p*_(i}_, *p*_(j}_ and *p*_(k)_ respectively.
µ	Specific growth rate (h^−1^).
*v* ^#^_tr(i)_, v*_tr(i)_	Number of transcripts of ORF_(i)_ per picogram of RNA substrate for cDNA synthesis.
*ω*′_aa(av)_, *ω*′′_aa(av)_	Specific protein synthesis rates (amino acid residues h^−1^) of population-average cells of reference and experimental cultures.

Theoretical values are shown by a prime and double prime which, respectively, denote values for reference and experimental cell cultures. Empirical values are shown by hashes and asterisks which, respectively, denote values for reference and experimental cell cultures.

Equation (I) defines exponential growth, during which a cell component *x*, such as RNA or protein,
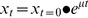
(I)


The specific synthesis rate ω_x_ of the component *x* is defined by equation (II) which is the differential of equation (I).
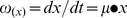
(II)


Equations (I) and to (II) apply to the term *n*_c–p(i)_ which defined as the gross number of copies of protein *p*_(i)_ per population-average cell. In other words, *n*_ c–p(i)_ is the number of times ORF_(i)_ was expressed during the lifetime of the cell.

Equation (III) is the appropriate form of equation (II) for the rate of synthesis of protein *p*_(i)_ encoded by ORF_(i)._

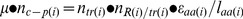
(III)


It follows from the equation for exponential growth that the left hand side of equation (III) is equal to the rate (copies h^−1^) of gene expression. The right hand side of the equation is equal to the product of the number *n*_R(i)_ of ribosomes translating *n*_tr(i)_ transcripts of ORF_(i)_ at any instant and the rate, 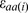 amino acid residues h^−1^, at which these ribosomes translate transcripts of ORF_(i)_. The conversion factor 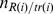 relates the number of transcripts of ORF_(i)_ with the number of ribosomes translating them; *l*_aa(i)_ amino acids is the length of protein *p*_(i)_.

Equation (III) provides the basis for our investigation because it defines the relation between the number of transcripts of ORF_(i)_ with the number of copies n_c–p(i)_ of p_(i)_ and it reveals that these two parameters are linked by the peptide chain elongation rate. Reports that the number of copies of a protein correlates poorly with the number of transcripts of ORF (see for example [15]) illustrate the need for quantitative analysis in order to obtain a better understanding of studies of ‘*omics*’ such as proteomics and transcriptomics.

To aid clarity ‘RESULTS AND DISCUSSION’ is divided into four sections. A summary of the equations used and the results of the microarray investigations are presented in the first section. The three sets of microarray data are presented as histograms which can be simulated by combining a small number of Gaussian distributions each corresponding to a particular *r*-value. The following section (**2 Analysis of microarray data and development of the theoretical framework** ***)*** shows how the microarray data for BCG-Pasteur and Msmeg provide the basis for further development of the theoretical framework described previously [13], [16]. The extended theory is presented in the METHODS section under the heading ‘Theoretical analyses’. Several features were considered, for example, standard deviations of *r*-values were estimated, constitutive gene expression was defined quantitatively and *r*-values for ORFs encoding ribosomal proteins were shown to measure the ratio of RNA to protein in the experimental culture divided by the ratio of the RNA to protein in the reference culture. An independent test of the theoretical framework was obtained by establishing methods for calculating the macromolecular compositions of population-average cells from measurements of the ratios DNA: RNA: protein.

The third section (**3 Comparisons of the effects of growth rate on the protein compositions of BCG-Pasteur and Msmeg**) describes the application of the extended theory to a comparison of the sets of microarray data reported for BCG-Pasteur and Msmeg in order to compare the changes in gene expression (and hence changes in the proteome) brought about by a change in growth rate. Changes in the protein compositions of the cell envelope and the cytoplasmic membrane were found to illustrate how bacteria adapt to growth conditions.

The ratios DNA: RNA: protein are available for both of the cultures of BCG-Pasteur investigated by microarray analysis and they provide further information about properties of population-average cells. These data are summarized in the fourth section (**4 Population-average cells of BCG-Pasteur**). An overview of all sections is presented in ‘CONCLUDING REMARKS’.

## Results and Discussion

### 1 Principal equations and presentation of microarray data

The symbols used in the mathematical analysis are presented in Table 1. The principal equations derived for the analysis of microarray data (see the ‘Theoretical Analyses’ section) are summarized in Table 2. The mathematical analysis was applied to two sets of microarray data published previously for two mycobacterial species; namely BCG-Pasteur [11] and Msmeg [12]. In both studies the bacilli were grown in a chemostat at two different rates. The genomic properties and growth conditions of the two species are summarized in Table 3, which shows that the genome of BCG-Pasteur is 62.6% of the size of the genome of Msmeg. BCG-Pasteur was grown at the slower rate of µ = 0.01 h^−1^ and at the faster rate of 0.03 h^−1^. Msmeg was grown at the same slower rate of µ = 0.01 h^−1^ and at the faster rate of µ = 0.15 h^−1^. In each case the faster growth rate stated is close to the maximum growth rate of the species concerned. An expression ratio (*r*-value) measures the expression of ORF_(i)_ in the experimental (slower growing) culture as a fraction of the expression of ORF_(i)_ in the reference (faster growing) culture (see Table 1).

**Table 2:** Equations used in the analysis of microarray data.

Equation Number	Equation	Comment
6	*r*_(i)_ ± σ = (n*_c–p(i)_/*n* ^#^_c–p(i)_) • (µ′′/µ′) • (ε′_aa(i)_/ε′′_aa(i)_) • (*m*′_RNA(av)_/*m*′′_RNA(av)_)	General equation for transcripts of ORF_(i)_: µ′≥ µ′′
9	*r*_(k)_ = (µ′′/µ′) • (*ε*′_aa(k)_/*ε* ′′_aa(k)_)	The gross abundance *n*_c–p(k)_ of protein *p*_(k)_ encoded by ORF_(k)_ is directly proportional to *m*_RNA(av)_.
11b	ε′_aa(k)_/ε′′_aa(k)_ = [(µ′/µ′′) +0.69]/1.69	The equation is linear when µ′≥ µ′′.
13c	*n* ^#^_c–p(i)_ = n*_c–p(i)_ • (<*r*_(k)_>/*r*_(i)_ ± σ) • (*m*′_RNA(av)_/*m*′′_RNA(av)_)	Derived from equation (6) by substitution of <*r*_(k)_> for (µ′′/µ′) • (*ε*′_aa(i)_/*ε*′′_aa(i)_) and rearranging.
21	*r*_(i)_ ± σ = (n*_c–p(i)_/*n* ^#^_c–p(i)_) • (*n*′_aa(av)_/*n*′′_aa(av)_)	Equation (21) is an alternative form of equation (13c) because (*n*′_aa(av)_/*n*′′_aa(av)_) = <*r*_(k)_> • (*m*′_RNA(av)_/*m*′′_RNA(av)_)

<*r*_(k)_>, denotes the average value found for 50 Zur independent ORFs encoding ribosomal proteins.

**Table 3:** Genomic properties and growth conditions of the bacterial species studied.

Property		BCG-Pasteur	Msmeg
Genome	Size (base-pairs)	4,374,522	6,988,209
	Pseudo genes	32	168
ORFs	Total number	4,033	6,938
	Protein coding	3,949	6,716
	Average size (base-pairs)	976	907
Growth conditions	Chemostat	carbon limited†	carbon limited^#^
	Faster growth rate	0.03 h^−1^	0.15 h^−1^
	Slower growth rate	0.01 h^−1^	0.01 h^−1^
	Temperature	37 °C	37 °C

† , [11]; ^#^, [12].

**Figure 1:**
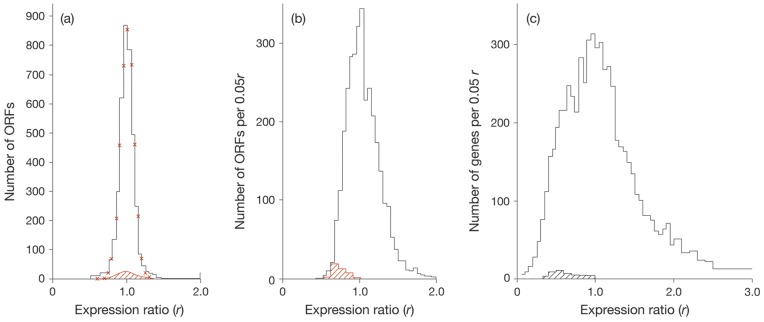
Histograms of expression ratios reported for *M.tuberculosis,* BCG-Pasteur and Msmeg. (a) Profile of expression ratios reported for *M.tuberculosis* grown in rolling bottles. The wild type (the reference culture) was compared with a *dosR* minus mutant (the experimental culture). Both cultures were found to grow at the same rate (a doubling time of about 17 h). The profile represents 3850 ORFs. The red crosses mark the Gaussian distribution calculated for 3850 expression ratios of *r* = 1.0±0.09. (b) Profile of expression ratios reported for cultures of BCG-Pasteur grown in a chemostat at growth rates of µ =  0.01 h^−1^ (the experimental culture) and µ = 0.03 h^−1^ (the reference culture). A total of 3475 expression ratios were reported [11]; the average value was found to be *r = *1.05±0.33. (c) Profile of expression ratios reported for cultures of Msmeg grown in a chemostat at growth rates of µ = 0.01 h^−1^ (the experimental culture) and µ = 0.15 h^−1^ (the reference culture). The complete profile comprised 6864 ORFs [12]. In all (a), (b) and (c) the hatched regions refer to 50 ORFs encoding for Zur-independent ribosomal proteins.

Up to 3,475 and 6,864 ORFs of BCG-Pasteur and Msmeg respectively were analysed. The numbers of ORFs found per r-value are summarized as histograms (see Fig. 1). Fig. 1a provides a reference profile for the study of the effects of growth rate on gene expression; namely, the profile found for wild type *M.tuberculosis* versus a *dosR* minus mutant [17]. Both cultures were found to grow at the same rate. Changes in growth rate led to broader profiles in BCG-Pasteur and Msmeg (see Fig. 1b and 1c). In all three cases the peak value was centred round *r* = 1.0 and one third or more ORFs were found to have values in the range *r* = 0.85–1.15. The histogram found for Msmeg (Fig. 1c) was broader than the profile found for BCG-Pasteur (Fig. 1b) with shoulders at *r* = 0.6 and *r* = 1.5 respectively. Figure 1 also shows the influence of the growth rate on the expression ratio of ribosomal proteins. The expression of the majority of ORFs encoding ribosomal proteins decreased (*r*_(i)_ <1.0) when mycobacteria grew at the slower rate (Figs. 1b and 1c).

### 2 Analysis of microarray data and development of the theoretical framework

The interpretation of the microarray data is based on equation (21) (see Table 2).

The expression ratio *r*_(i)_ of ORF_(i)_ was shown to be directly proportional to the relative concentrations of the encoded protein *p*_(i)_ in population-average cells of the experimental and reference cultures (see equation 21, Table 2). This equation includes the term (*n*′_aa(av)_/*n*′′_aa(av)_) which has a numerical value in BCG-Pasteur of 1.56 (see Table 4). The use of the numerical value allows the ratio of the number of copies of the encoded protein to replace the expression ratio in Fig. 1b. The simulated profile shows that the relative numbers of copies of the encoded protein are centred on 0.64 (the reciprocal of 1.56). The average value for 3475 ORFs was found to be n*_c–p(i)_/*n* ^#^_c–p(i)_  = 0.69±0.22. Thus the results obtained from microarray analysis benefit from the chemical data for the protein contents of experimental and reference cells of BCG-Pasteur (Table 4).

**Table 4:** Macromolecular compositions of population-average cells of *M.bovis* BCG Pasteur grown in a chemostat§.

Property	µ′′ = 0.01 h^−1^	µ′ = 0.03 h^−1^
Genomes per cell¶	1.31	1.34
DNA (fg) per cell, *m*_DNA(av)_.	6.30	6.44
Protein per cell		
mass (fg), *m*_p(av)_.	44.15	69.00
amino acid residues (*n*_aa(av)_)‡.	2.47×10^8^	3.86×10^8^
RNA (fg) per cell, *m*_RNA(av)_.	2.62	9.53
Ribosomes per cell (*n*_R(av)_)	1020	3730
Fraction of ribosomes synthesizing proteins†	0.80	0.80
Specific protein synthesis rate (amino acid residues per cell h^−1^)	2.47×10^6^	11.55×10^6^
Peptide chain elongation rate (amino acid residues per ribosome h^−1^) *ε*_aa(av)_	3.03×10^3^	3.90×10^3^
Dry mass (fg) per cell, *m*_dc(av)_	177.6	256.9
*m*_p(av)_/*m*_dc(av)_	0.25	0.26

§ , The table is based on the ratios DNA: RNA: protein : dry cell mass reported for the two cultures of *M. bovis* BCG Pasteur [21] which were later investigated by microarray analysis [11].

¶ , The numbers of genome equivalents per population-average cell were obtained by the methods described in Supporting data for Table 4 (Material S1).

‡ , *n*_aa(av)_ was calculated from *m*_p(av)_ on the basis that 1 fg protein is equal to 5.6×10^6^ amino acid residues [19].

† , At any instant during exponential growth about 80% of ribosomes are engaged in peptide bond formation [19].

Up to 3,448 ORFs of BCG-Pasteur were found to have expression ratios in the range 0.42–2.00 (See Fig. 1b). We infer that at least 1,750 ORFs with *r*-values in the range 0.85–1.15 encode proteins that have the same concentrations at both growth rates; these ORFs are designated constitutive. The concentrations of the proteins encoded by many of the 720 ORFs with *r-*values in the range 0.42–0.85 were increased up to two-fold on increasing the specific growth rate from µ = 0.01 h^−1^ to µ = 0.03 h^−1^. Finally, the concentrations of proteins encoded by many of the 978 ORFs with *r*-value*s* in the range 1.15–2.00 were up to twofold higher in the slower growing culture.

The effects of a fifteen fold change in growth rate on the expression of ORFs of Msmeg led to *r*-values ranging from 0.085 to 50.7 (see Fig. 1c). In other words, the ratio of the concentrations of the encoded protein ranged from 0.085–50.7 as a result of changing the growth rate. The *r*-value*s* reported for Msmeg were distributed around the peak values of *r* = 1.0; with 1714 ORFs within the range 0.85–1.15 (therefore defined as constitutively expressed). Approximately 1300 ORFs were down regulated (*r*<0.5) and approximately 900 ORFs were up regulated more than twofold (*r*>2.0).

#### 2.1 The accuracy of microarray measurements is defined by the standard deviation

A crucial factor of the experimental approach is the fidelity with which cDNA preparations used in microarray analysis reflects the compositions of the RNA components within cognate population-average cells. Although this factor is difficult to measure directly it is likely to contribute to the standard deviations that define the profiles shown in Figure 1. A provisional target of ±0.10 is proposed on the basis of results (see Fig. 1a) obtained when the gene expression of wild type *M.tuberculosis* was compared with a *dos*R minus mutant (as described by [17]). This result sets the standard that can be achieved in the analysis of microarray data.

**Figure 2:**
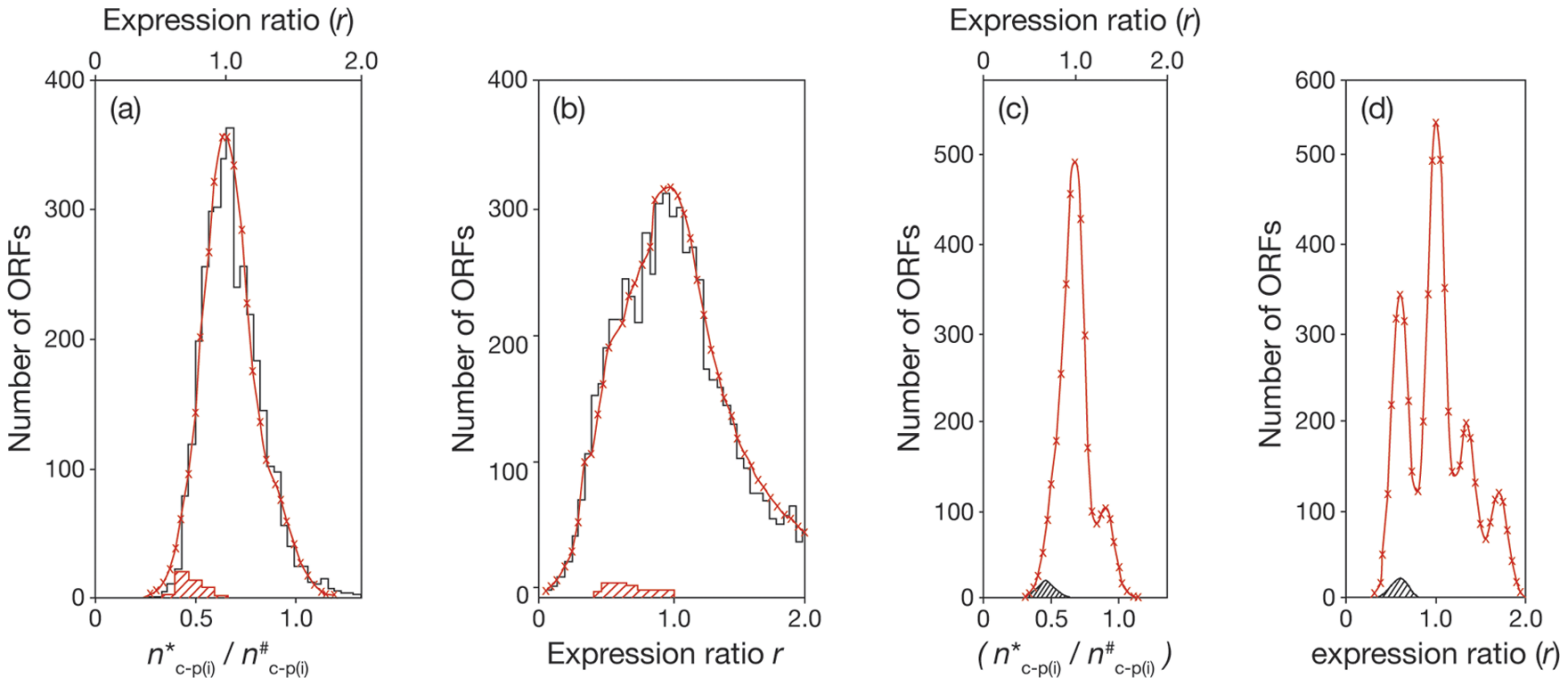
Comparisons of observed and simulated profiles of expression ratios reported for BCG-Pasteur and Msmeg. The experimental results are shown in black and the simulated profiles in red with crosses marking calculated values. (a) BCG-Pasteur. The calculations for the simulated profile were based on the assumption that the expression ratios of each of the proposed subgroups of ORFs conform to a normal Gaussian distribution with a standard deviation of ±0.15. The dominant subgroup (ORF_(j)_ *r*_(j)_  = 1.0±0.15) comprised 2350 constitutively expressed genes. The second subgroup (ORF_(k)_) comprised 100 ORFs (*r*_(k)_  = 0.70±0.15). The third subgroup (ORF_(l)_) comprised 500 ORFs (*r*_(l)_  = 0.80±0.15). The fourth subgroup (ORF_(m)_) comprised 500 ORFs (*r*_(m)_  = 1.45±0.15). Thus 3450 genes were considered. The average expression ratio was found to be *r* = 1.02 per ORF. The distribution of ORFs according to the relative number (n*_c–p(i)_/*n* ^#^_c–p(i)_) of copies of protein *p*_(i)_ in experimental and reference cultures was shown. (b) Msmeg. The simulated profile was calculated for 6180 ORFs in the range *r = *0.0–2.0 comprising five subgroups conforming to a normal Gaussian distribution, each with standard deviation of ±0.20. The compositions of the subgroups were as follows: 2750 constitutively expressed ORFs (*r*_(j)_  = 1.0±0.20); 1750 ORFs (*r*_(a)_  = 0.60±0.20), which includes ORFs of the subgroup ORF_(k)_ (*r*_(k)_  = 0.60±0.20); 1000 ORFs (*r*_(b)_  = 1.35±0.20); 600 ORFs (*r*_(c)_  = 1.70±0.20) and 350 ORFs (*r*_(d)_  = 2.30±0.20). In all, 6450 ORFs were considered. (c) Refined simulated expression profiles for BCG-Pasteur. (d) Refined simulated expression profiles for Msmeg. The simulated profiles shown in (a) and (b) were recalculated in (c) and (d) by changing the standard deviations to ±0.1.

Accordingly, the simulated profiles (see Figs. 2(a) and 2(b)) were further refined (see Figs. 2(c) and 2(d) by using the appropriate combinations of subgroups of ORFs but assigning a value of ±0.10 throughout for the standard deviation. The profiles presented in Figures 2c and 2d may be regarded as examples of results obtained for ‘best achievable’ cDNA preparations. Comparison of Figures 2a and 2b respectively with 2c and 2d shows that resolution is lost as the standard deviation increases.

The similarity of the observed and simulated profiles led to the following conclusions. First, the standard deviations of ±0.15 and ±0.20 provide independent estimates of the accuracies of the two sets of microarray measurements studied; secondly, as shown previously [13], the standard deviation found for *r*-values of ORFs encoding ribosomal proteins was a useful starting point for the analysis; thirdly, irrespective the total number of ORFs per genome, the number of constitutively expressed genes was found to be similar for both BCG-Pasteur (2350 or 59% of ORFs) and Msmeg (2750 or 49% of ORFs)); fourthly, many features of the profile can be accounted for by the properties of a small number of subgroups of ORFs.

#### 2.2 Subgroups of ORFs and their significance

The profiles shown in Figure 1 reveal that in each study at least one third of ORFs had expression ratios of 1.00±0.15 and it is convenient to consider them as a subgroup ORF_(j)_ of the general group ORF_(i)_. The significance of *r* = 1.0 is that the number of copies *n*_c–p(j)_ of the encoded protein *p*_(j)_ is directly proportional to cell size (see the Theoretical Analyses). Equation (15) (see Theoretical Analysis) shows that the concentration of *p*_(j)_ measured by the ratio *n*_c–p(j_)/*n*_aa(av_) is maintained constant irrespective of growth rate. Since *r*_(j)_ = 1.0, then rearrangement of equation (21) leads to equation (21a).
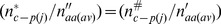
(21a)


The finding that a high proportion of ORFs are constitutively expressed allows further analysis of the histograms shown in Figure 1 on the basis of the assumption that the standard Gaussian distribution applies to each subgroup such as ORF_(j)_ and ORF_(k)_.

The height of the histogram, which is governed by both the number of ORFs in the subfamily and the standard deviation of the expression ratios, provides the basis for further analysis of the observed profiles (see Fig. 1). The reference profile reported for the comparison of wild type *M.tuberculosis* and a *dosR* minus mutant (see Fig. 1a) was found to be simulated by a single component calculated for 3850 ORFs with an *r*-value of 1.00±0.09. The results obtained for BCG-Pasteur are shown in Figure 2a. The observed histogram comprised 3448 ORFs with *r*-values less than 2.0. The simulated version was constructed from 3450 ORFs comprising four subgroups (see legend to Fig. 2). The best fit was obtained with a standard deviation of ±0.15 for each subgroup.

The observed and simulated profiles obtained for Msmeg are compared in Fig. 2b. More than 6100 ORFs were compared, covering *r*-values ranging from 0.1 to 2.0. The simulated version was based on six subgroups (see legend to Fig. 2). The best fit was obtained with a standard deviation of ±0.20 for each subgroup.

The subgroups ORF_(a)_ (which includes ORF_(k)_), ORF_(j)_ and ORF_(b)_ of Msmeg are similar respectively to subgroups ORF_(k)_, ORF_(j)_ and ORF_(m)_ of BCG-Pasteur. In each case, the subgroup ORF_(j)_ comprises constitutively expressed genes. The subgroup ORF_(k)_ comprises genes encoding proteins related to ribosome structure and function. In contrast, the subgroups ORF_(c)_ and ORF_(d)_ were found only in Msmeg, and the subgroup ORF_(l)_ was only found in BCG-Pasteur (see legend to Fig. 2).

#### 2.3 Expression ratio of ORFs members of the Zur regulon

Only 27 of the 3,475 ORFs of BCG-Pasteur that were examined were found to have *r*-values in the range 2.00–7.69. Fourteen of them, including five encoding ribosomal proteins, are now known to be under the control of Zur the Zinc related regulon [18] which regulates a total of 32 genes of BCG-Pasteur. These Zur regulated genes are listed in Table S1 (supporting data) and the effects of growth rate and zinc deficiency on gene expression are compared. Table S1 reveals that in the slower growing culture 25 of the 32 genes were up regulated with fourteen of them having expression ratios greater than 2.00, as mentioned previously. This comparison suggests that the Zur regulon was influential at the slower growth rate.

Four of the above-mentioned five Zur-dependent ribosomal proteins are unusual because each is encoded by two genes. With these four exceptions, each of the mycobacterial ribosomal proteins is encoded by a single gene. The exceptions are rpsN, rpsR, rpmB and rpmG, each of which is encoded by two closely related but non-identical genes (for discussion see [13]). One operon, which is under the control of Zur comprises rpsR2, rpsN2, rpmG1 and rpmB2; the gene encoding rpmB1 is also controlled by Zur (see Table S1). Contrary to their Zur-dependent counterparts, all other ORFs encoding ribosomal proteins were down regulated at the slower growth rate (see below and Figs. 1b and 1c).

Those ORFs (MSMEG_6065 to MSMEG_6068) encoding the subset of ribosomal proteins regulated by Zur were found to have *r*-values in the range 0.85–1.15 compared with the up regulation of the orthologous ORFs (BCG_2074c – BCG_2077c) of BCG-Pasteur (Table S1). We have yet to find an explanation for the different responses of these Zur-regulated genes comparing the two species.

#### 2.4 Expression ratios of ORFs encoding ribosomal proteins

Ribosomal proteins are regarded as representative of a subgroup (namely ORF_(k)_) of ORF_(i)_ that encode a protein whose abundance is directly proportional to *m*_RNA(av)_, for example subunits of ATP synthase and aminoacyl-tRNA synthases (see Table 5).

**Table 5:** Expression ratios of ORF_(k)_ encoding protein, *p*_(k)_, whose gross abundance is directly proportional to the RNA content of population-average cells.

Gene family	Average Expression ratios (<r*_(k)_*>)	
	BCG-Pasteur	M.smeg
Ribosomal proteins	0.71±0.10	0.62±0.25
ATP synthase (subunits)	0.75±0.10	0.53±0.11
Aminoacyl tRNA synthases	0.88±0.13	0.59±0.22

The ratio (µ′′/µ′) differs in the two sets of microarray studied; namely, threefold in the case of BCG-Pasteur and fifteen fold in the case of Msmeg. The number of ribosomes per population average cell increases with increasing growth rate [19]. Hence, the changes in the number of ribosomes per population-average cell would be expected to be much larger for a fifteen-fold change than for a threefold change in growth rate. However, the expression ratios reported for the 50 ORFs encoding ribosomal proteins, which are not under the control of Zur, were found to be very similar for the two sets of microarray data (see Figs. 1b and 1c and Table 6). An explanation of this unexpected result lies in the design of microarray experiments; analysis is based on comparisons of the numbers of transcripts per unit mass of RNA rather than on comparisons of the numbers of transcripts per population-average cell. Thus, equal masses of RNA will correspond to equal numbers of ribosomes, which are thought to account for more than 80% of the RNA content of a cell (see for example [19]).

**Table 6:** Comparison of properties of BCG-Pasteur derived from microarray studies with properties derived from the ratios DNA: RNA: protein: dry cell mass.

	Values calculated	
Property	Microarray analysis	DNA : RNA : protein§
(µ′′/µ′)	0.33	0.33
(*ε*′_aa(av)_/*ε*′′_aa(av)_)	2.18	1.29 (2.18)
<*r*_(k)_>	0.71	0.43 (0.71)
(*n*′_R(av)_/*n*′′_R(av)_)	2.20 †	3.64 (2.20)
<*r*_(k)_>•(*m*′_RNA(av)_/*m*′′_RNA(av)_)	1.53	1.55
(*n*′_aa(av_/*n*′′_aa(av)_)	1.56‡	1.56
(*m*′_dc(av)_/*m*′′_dc(av)_)	na	1.45

§ , See Table 4.

† , Calculated using the equation in the shown comment to equation (21) (see Table 2) on the basis of the assumption that *m*′_p(av)_/*m*′′_p(av)_  = 1.56

‡ , Calculated using the equation in the shown comment to equation (21) (see Table 2) on the basis of the assumption that *n*′_R(av)_/*n*′′_R(av)_  = 2.20.

na, not accessible from microarray data. Preferred values are enclosed in brackets.

With few exceptions a mycobacterial ribosomal protein is encoded by a single ORF per genome as discussed previously [13]. Each ribosomal protein is thought to be located mainly in ribosomes with few copies (<2%) found in the cytoplasm as was shown for *E. coli* [20]. Thus, the number of copies *n*_c–p(k)_ of ribosomal protein *p*_(k)_ is directly proportional to the number of ribosomes per cell and hence the mass of RNA (*m*_RNA(av)_) of the population-average cell.

The definition of *r_(k_)* is stated in the comment to equation 21 in Table 2 and in the discussion of equation 20 (see Theoretical Analyses) as the ratio of RNA to protein in experimental cultures to the ratio of RNA to protein in reference cultures. This relation is manifest in different ways.

**Figure 3:**
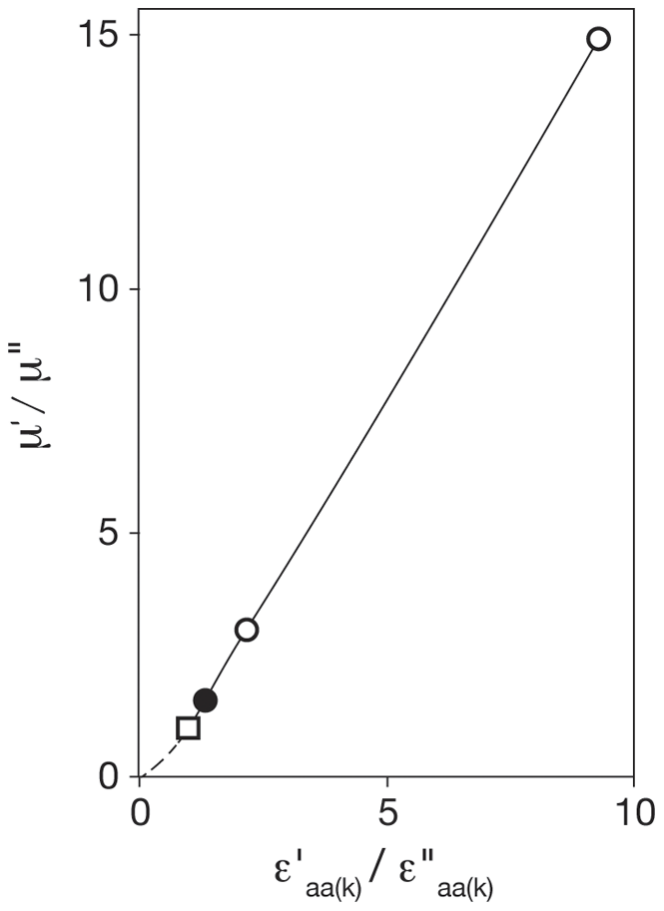
The relation of the ratios of the specific growth rates (µ′/µ′′) to the ratios of the peptide chain elongation rates (*ε*′_aa(k)_/*ε*′′_aa(k)_) of ribosomal proteins. Values of (*ε*′_aa(k)_/*ε*′′_aa(k)_) were evaluated using equation (9), Table 2. The plot is linear when µ′>µ′′ (see equation (11b), Table 2). The reference point (µ′/µ′′)  =  (*ε*′_aa(k)_/*ε* ′′_aa(k)_)  = 1.0 is shown as a square; the open circles refer to BCG-Pasteur and Msmeg; the filled circle refers to *E. coli* B/r (see Table 3, [19]). The broken line applies when µ′< µ′′.

The expression ratios presented in Table 5 are in accord with equation (9), the simplified form of equation (6), for proteins within subgroup ORF_(k)_ (see Table 2). Both *µ*′ and µ′′ and average values of *r*_(k)_ are known for each of the studies, allowing *ε*′_aa(k)_/*ε*′′_aa(k)_ to be evaluated. The empirical plot of (µ′/µ′′) against (*ε*′_aa(k)_/*ε*′′_aa(k)_) was found to be linear when µ′ is greater than µ′′ (see Fig. 3), in accord with equation (11a) (see Theoretical Analyses). Implicit in equation (11a) is the notion that when µ′ exceeds µ′′ then *ε*′_aa(k)_ exceeds *ε*′′_aa(k)_.

Formally, equation (11a) was derived for ORF_(k)_ but we infer that same relationship between (µ′/µ′′) and (*ε*′_aa(k)_/*ε*′′_aa(k)_) applies to all ORFs under consideration. We considered the hypothesis that equation (11a) applies to all exponentially growing bacteria when the specific growth rate exceeds the time needed to replicate the genome. Then, at least to a first approximation, the mass of DNA per cell is independent of growth rate. This possibility was found to be supported by the available data for *Escherichia coli* B/r [19] as discussed in supporting data for equations (11a and b).

The data available for the two cultures of BCG-Pasteur [21] include measurements of their macromolecular compositions derived from the ratios DNA: RNA: protein (Table 4) which allow *r*_(k)_ and *ε*′_aa(av)_/*ε*′′_aa(av)_ to be evaluated independently of microarray measurements. The two sets of data (microarray and macromolecular composition, see Table 6) were found to agree within 40%, which appears satisfactory in view of the diversity of the methods used and the assumptions made.

The ratio of the RNA content measured by chemical analysis (see Tables 4 and 6), *m*′_RNA(av)_/*m*′′_RNA(av)_  = 3.64, is unlikely to be accurate because it leads to the ratio *ε* ^#^_aa(k)_/ε*_aa(k)_  = 1.29 which does not agree with the data presented in Figure 3. The value (2.20) of the ratio of the RNA contents derived from *r*-values found for ribosomal proteins (see Table 6) is much closer to the guide value of 2.55 obtained on the basis of the assumption that the specific protein synthesis rate is proportional to the third power of the RNA: protein ratio [16].

Equation 21 (Table 2) was used to evaluate the average value of the ratio *n* ^#^_c–p(k)_/*n* ^*^_c–p(k)_ for ribosomal proteins. Substitutions were made for *r*_(i)_  = < *r*_(k)_>  = 0.71 and, as shown in the preceding paragraph, for n*_c–p(i)_/*n* ^#^_c–p(i)_  = 0.64 leading to (*n* ^#^_c–p(k)_/*n* ^*^_c–p(k)_)  = 2.20. We assume that the ratio of the ribosomal proteins is equal to the ratio of the RNA contents; that is, (*m*′_RNA(av)_/*m*′′_RNA(av)_)  = 2.20. We regard this value to be more reliable than the ratio of 3.64 obtained by chemical analysis (see Tables 4 and 6) because it is based on an average value, < *r*_(k)_ >, which is based on 50 independent measurements. We conclude that the chemical and microarray data reinforce one and other. The inclusion of empirical values for peptide chain elongation rates would strengthen the chemical data. The peptide chain elongation rates shown in Tables 4 and 6 were calculated from the RNA and protein contents of population-cells and so reflect any errors in these measurements.

### 3 Comparisons of the effects of growth rate on the protein compositions of BCG-Pasteur and Msmeg

#### 3.1 ORFs constitutively expressed at different growth rates

Expression ratios of selected constitutive genes (members of the subfamily ORF_(j)_) of BCG-Pasteur and Msmeg that encode proteins needed for DNA replication and repair are shown in Table 7. With one exception, the expression ratios of genes encoding proteins required for DNA replication and repair were found to fall within the range 0.71–1.24; the average value was 0.92±0.16 after a fifteen fold change in the growth rate. We infer that the efficient replication and repair of DNA is achieved by maintaining a near to constant concentration of the appropriate enzymes. In contrast, the substrate, DNA, was present throughout at approximately 1.4 genome equivalents per population-average cell.

**Table 7:** T**able 7.** Examples of genes (ORF_(j)_) regulated by cell size (constitutive expression) ¶.

		BCG-Pasteur (H37Rv)		Msmeg	
gene		locus tag^§^	r_(j)_ value	locus tag	r_(f)_ value
ORFs involved in DNA replication					
*par*B	BCG_0023c (Rv3917c)		0.90	MSMEG_6938	1.25
*par*A	BCG_0024c (Rv3918c)		0.96	MSMEG_6939	0.74
*dna*A	BCG_0031 (Rv0001)		nr	MSMEG_6947	0.98
*dna*N	BCG_0032 (Rv0002)		0.75	MSMEG_0010	0.89
*dna*J1	BCG_0390 (Rv0351)		nr	MSMEG_4504	0.89
*din*X	BCG_1589 (Rv1537)		1.06	MSMEG_3172	0.87
*dna*E1	BCG_1600 (Rv1547)		0.99	MSMEG_3178	0.91
	BCG_2429c (Rv2413c)		1.18	MSMEG_4572	0.82
*ssb*	BCG_2498 (Rv2478c)		1.00	MSMEG_4701	1.05
*dna*E2	BCG_3442c (Rv3370c)		1.71	MSMEG_1633	0.95
*dna*Q	BCG_3771c (Rv3711c)		1.06	MSMEG_6275	1.25
	BCG_3781c (Rv3721c)		1.46	MSMEG_6285	0.47
	Not in BCG Pasteur (Rv1985c)			MSMEG_0548	0.96
ORFs involved in cell wall formation					
*mur*A	BCG_1376 (Rv1315)		0.89	MSMEC_4932	0.95
*mur*I	BCG_1400 (Rv1338)		1.06	MSMEC_4903	1.01
*fts*Z	BCG_2167c (Rv2150c)		1.00	MSMEG_4222	1.09
*fts*K	BCG_2498 (Rv2478c)		0.88	MSMEG_2690	0.93
ORFs involved in DNA repair					
*rec*F	BCG_0033 (Rv0003)		0.89	MSMEG_0003	0.86
*rec*D	BCG_0675c (Rv0629c)		1.38	MSMEG_1325	0.83
*rec*B	BCG_0677c (Rv0630c)		nr	MSMEG_1327	1.06
*rec*C	BCG_0678c (Rv0631c)		1.43	MSMEG_1328	0.81
	BCG_1217 (Rv1156)		0.70	MSMEG_5156	1.05
*rec*N	BCG_1734 (Rv1696)		1.38	MSMEG_3749	0.91
	BCG_2136 (Rv2119)		1.00	MSMEG_3907	0.93
	BCG_2744 (Rv2731)		0.89	MSMEG_2731	1.13
*rec*X	BCG_2749c (Rv2736c)		1.15	MSMEG_2724	0.71
*rec*A	BCG_2750c (Rv2737c)		0.87	MSMEG_2723	1.18
*din*B	BCG_3081 (Rv3056)		1.34	MSMEG_2294	1.07
*rad*A	BCG_3650 (Rv3585)		1.27	MSMEG_6079	0.80
*rec*Q	Not applicable			MSMEG_5397	0.85
Other ORFs regulated by cell size					
*rpo*B	BCG_0716 (Rv0667)		1.11	MSMEG_1367	1.33
*rpo*C	BCG_0717 (Rv0668)		0.90	MSMEG_1368	0.96
	BCG_2228 (Rv2212)		1.22	MSMEG_4279	0.94
*nus*B	BCG_2555c (Rv2533c)		1.14	MSMEG_3036	0.86
*nus*A	BCG_2861c (Rv2841c)		0.93	MSMEG_2625	1.10

¶ , see equation (11) for definition; ^§^, the loci numbers corresponding to *M. tuberculosis* H37Rv are also indicated between brackets, as it appears in [11]; nr, no result.

*Toxin/antitoxin systems*: Toxin/antitoxin systems are present in the majority of bacteria including mycobacteria [22]. These systems are considered to be involved in ensuring that a small proportion of cells survive (‘persist’) by entering a dormant state when stressed by conditions such as exposure to antibiotics. It is thought that a membrane-acting polypeptide sends a cell into a dormant state by decreasing its energy supply; that is by reducing ATP levels and decreasing the proton-motive force [23]. The *r*-values reported for the toxin/antitoxin systems of the two mycobacterial species were examined. It was found (see Table S2) that the *r*-values of 42 ORFs of BCG-Pasteur encoding components of toxins/antitoxins [22], [24] had an average value of 0.90±0.12. Msmeg has only three toxin/antitoxin systems showing an average *r*-value of 0.95±0.22 which are also constitutively expressed [25]. Both BCG-Pasteur and Msmeg grew exponentially at the slower growth rate which we regard as the normal response to a poorer supply of nutrients. We infer that the toxin/antitoxin systems provide the cell with protection against stress but play no part in the adjustment to slower growth.

#### 3.2 The interface between the cell and its environment

The bacterial cell wall forms the outer permeability barrier and provides the interface between the cell and its environment and so its composition may be expected to be dependent on growth rate. We explored the influence of the growth rate on the composition of the cell envelope, by examining the *r*-values of genes related to signalling and transport across the cell envelope.

Constituents of the cell envelope include the PE, PE-PGRS and PPE families of proteins which are known to be abundant in pathogenic mycobacteria and scarcely represented in non pathogens [26]; water-filled porin or porin-like channels allow a hydrophilic solute to diffuse through the cell wall, into the periplasmic space, before it is actively transported across the cytoplasmic membrane into the cytoplasm. One function of the cytoplasmic membrane is the generation of energy (for reviews see [27], [28]); for example, the formation of a peptide bond requires the participation of four high-energy phosphate bonds commonly supplied by ATP. Copies of ATP synthase are located within the cytoplasmic membrane. Another function is the regulation of both the influx and efflux of metabolites. This function is achieved through several signal transduction systems that include two component regulatory systems (for review see [29], [30]), ATP-binding cassette transporter proteins (for review see [31]) and serine/threonine protein kinases [32]–[34]. The influences of growth rate on the expressions of genes encoding the above-mentioned components are described below.

*PE, PE_PGRS and PPE proteins* The cell envelopes of BCG-Pasteur and Msmeg differ in their compositions, in particular, in the numbers of members of the PE, PE_PGRS, PPE families of proteins. These proteins are considered to be located in the outer membrane [35]–[37]. For example, BCG-Pasteur has 33 PE, 62 PE_PEGRS and 61 PPE. In contrast, Msmeg has six PE, no PE_PGRS and six PPE. The histograms shown in Figure 1S reveal that changing the growth rate of BCG-Pasteur altered the gross concentrations of individual members of the PE, PE_PGRS and PPE families, as shown by the more than twofold range of expression values from 0.70 to 1.80.

*Porins:* Porins are important components of the outer membrane because they form aqueous channels that allow hydrophilic metabolites present outside the cell to diffuse into the periplasmic space.

The structure of MspA (MSMEG_0965) one of the four porins of Msmeg was established by X-ray crystallography [38]–[40]. As shown in Table S3, concentrations of three of its four porins increased as the growth rate decreased thereby increasing the permeability of the slower growing cell to hydrophilic solutes. MspA-like proteins have yet to be found in members of the *M.tuberculosis* complex, including *M. bovis* BCG. Evidence was obtained [41] for a pore-forming protein OmpATb (Rv0899; corresponding to BCG_0951, *r* = 0.90) and a systematic study [37] has identified other candidates; for example, Rv1698 (BCG_1736, *r* = 0.79). Neither of these candidates for porin proteins in BCG-Pasteur appears to increase their concentrations appreciably in relation to growth rate.

*ATP synthases*: Two studies of the abundance of mRNA species reported that mRNAs encoding subunits of ATP synthase were at least as abundant as mRNAs encoding ribosomal proteins [42], [43]. These reports support the inference that the numbers of copies of ATP synthase and the number of ribosomes are similar. Accordingly, as shown in Table 5, the number of copies of ATP synthase varies according to the number of ribosomes.

**Figure 4:**
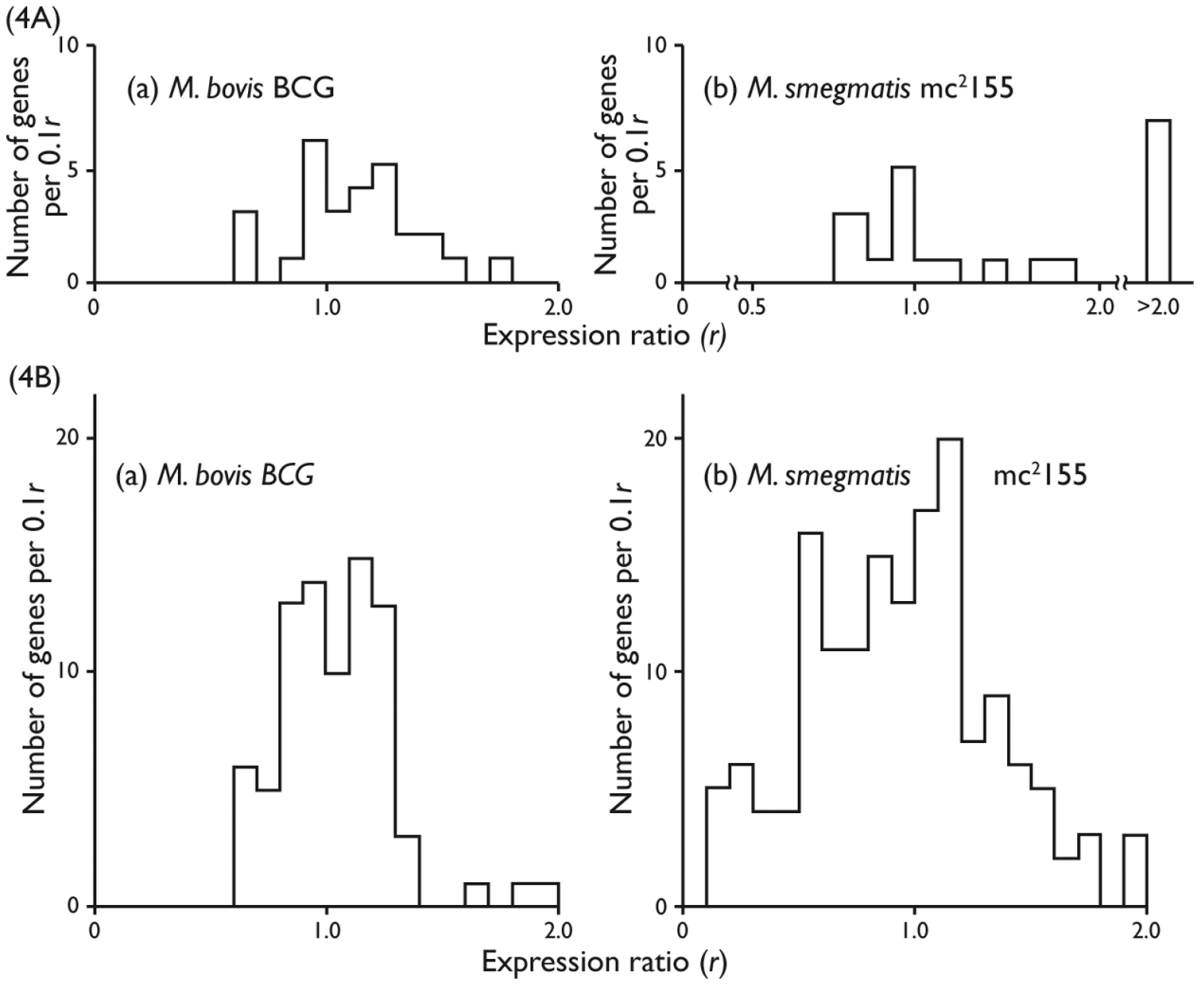
Effects of growth rate on the expressions of ORFs encoding proteins involved in the bacterial outer permeability barrier. (A) Two Component systems proteins of the BCG-Pasteur (a) and Msmeg (b). (B) ATP binding casette proteins of BCG-Pasteur (a) and Msmeg (b).

*Two component regulatory systems:* Traditional slow growing mycobacteria have many genes encoding components of two component regulatory systems [29]. With two exceptions, the expression ratios of the appropriate genes of BCG-Pasteur (see Table S4) ranged from r = 0.67 to r = 1.44 as a result of the threefold change in growth rate; in general, expression could be said to be either constitutive or modestly increased or decreased as the growth rate decreased (Fig. 4A(a) and Table S4). Properties of two component regulatory systems of Msmeg are shown in Fig. 4A(b). The number of genes was found to be 42 compared with 31 found for BCG-Pasteur. Up to 8 paired systems and two unpaired components were common to both species (see Table S4 (a)). Another 22 genes were found to be characteristic of fast growers (see Table S5). Expression ratios were found to cover a wider range in Msmeg than in BCG-Pasteur; namely, from *r* = 0.36 to *r*>3.0 (see Tables S4 and S5). This result is attributed to the fifteen-fold change in growth rate compared to the three fold change in BCG-Pasteur.

The results show that the gross concentrations of several two component systems vary with growth rate, probably to regulate the active transport of metabolites across the cytoplasmic membrane. Thus, it is likely that the number of copies of a particular system, per unit area of membrane, may vary with growth rate.

*ATP binding cassette (ABC) proteins:* ABC proteins are involved in the transport (both influx and efflux) of substances ranging from small ions to large polypeptides across the cytoplasmic membrane (for reviews see [31], [44]).

The genome of BCG-Pasteur was found to encode 88 components of ABC transporters, in common with other slow growers. The three fold change in growth rate led to *r*-values ranging from 0.6 to 2.0 (see Table S6 and Fig. 4B(a)); 56 genes were found to have values in the range 0.8 to 1.2 (constitutively expressed).

A total of 283 genes encoding components of ABC transporters (see Tables S6, S7 and Fig. 4B(b)) were identified in Msmeg; 73 of them were orthologous of BCG-Pasteur genes (identified in Table S6 (a)). The fifteen fold change in growth rate led to changes in *r*-values ranging from 0.1 to 4.5 (Fig. 4B(b)); 88 genes (38%) were found to have values in the range 0.8 to 1.2, compared with 56 genes (64%) found for BCG-Pasteur. Thus, the data show that the genome of Msmeg encodes almost three times the number of components of ABC transporters than the genome of BCG-Pasteur. Comparison of values of Table S6 (a) reveal that the fifteen fold change in the growth rate of Msmeg led to more extensive changes in *r*-values than the three fold change in the growth rate of BCG-Pasteur.

*Serine threonine protein kinases (STPKs):* STPKs are also components of the cytoplasmic membrane which play a key role in regulating key metabolic processes including the regulation of the growth cycle, development and responses to stress [32]. These authors identified eleven STPKs in BCG-Pasteur and at least 13 in Msmeg (Table S8). Orthologues of six STPKs were found in both species. The *r*-values of STPKs of BCG-Pasteur were found to range from 0.67 to 1.51 compared with the broader range from 0.69 to 4.34 found for STPKs of Msmeg which is attributed to the fifteen-fold change in growth rate in Msmeg compared to the three fold change in BCG-Pasteur.

### 4 Population-average cells of BCG-Pasteur

**Figure 5:**
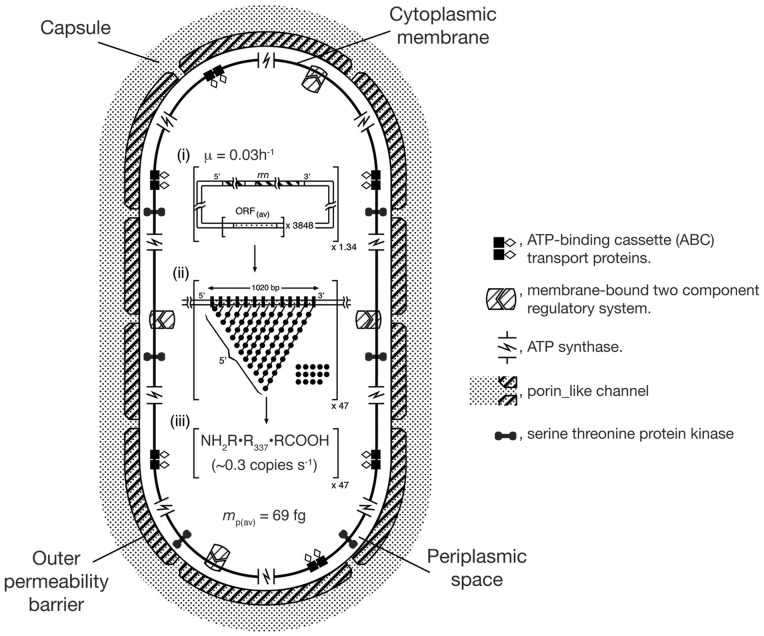
A schematic view of a population-average cell of BCG-Pasteur (*t*_d_  = 23 h) illustrating features important on growth including transcription/translation activities. The cell is represented as a cylinder with hemispherical ends (axial ratio of 1:2). Transcription/translation activities are based on µ = 0.03 h^−1^, *n*R_(av)_  = 3730 ribosomes and *m*p_(av)_  = 69 fg protein per population-average cell (see Table 4). Porins are shown as Y-shaped channels traversing the outer membrane permeability barrier. (i). Summary of genomic properties per population-average cell (see Tables 3 and 4). The properties of the genome; namely the number of *rrn* operons (solid bars) and the number of ‘average’ ORFs are (stippled bars) presented within the square brackets. The number of genome equivalents is indicated outside the brackets on the lower right hand side. (ii)Transcription/translation activity of an ‘average’ ORF is represented schematically within the square brackets in the form of a fibril diagram. The size (bp) of an ‘average’ ORF is shown and also the locations of the 5′-ends of nascent mRNA transcripts. Nascent polypeptide chains are not shown. The proportion of non-programmed ribosomes per ORF is indicated by the relative number of free ribosomes. The number of ‘average’ ORFs being transcribed/ translated at any instant is shown by the number outside the square brackets on the lower right hand side; vertical. Black bars along the ORF represent RNAP holoenzymes; filled circles represent ribosomes; and the lines joining these circles represent nascent mRNA. (iii)Rate of synthesis of ‘average’ proteins. The rate of synthesis per fibril is indicated within the square brackets. The number of fibrils synthesizing protein is given outside the square brackets on the lower right-hand side. The product of the two numbers provides the specific protein synthesis rate (amino acid residues h−1). Sections (i), (ii) and (iii) are based on Cox [16].

A schematic view of a population-average cell of BCG-Pasteur grown at the faster rate (with a doubling time of 23 h) is presented in Figure 5. The Figure shows quantitatively both the macromolecular composition and the overall protein synthetic activity of the population average cell. Features of the outer permeability barrier and cytoplasmic membrane are symbolic because quantitative data for the numbers of copies of individual components per cell are not available.

Several features of our study lead to an overall view of the changes in the proteome of BCG-Pasteur owing to a change in the specific growth rate.

First, the mathematical framework relates the expression ratio with the ratio (n*_c–p(i)_/*n* ^#^_c–p(i)_) of the gross number of copies of the encoded protein (see equation (21), Table 2). Secondly, the ratio (n*_c–p(i)_/*n* ^#^_c–p(i)_) may be evaluated once the ratio (*n*′_aa(av_/*n*′′_aa(av)_) is known (see Table 4). Thirdly, ORFs were divided into several subgroups according to their expression ratios (Fig. 2a and 2b).

These considerations enable the compositions of the proteomes of experimental and reference cells to be compared (see METHODS section 2 Changes in the protein moiety of BCG-Pasteur with growth rate inferred from microarray data).

Each of the 3475 ORFs investigated was expressed in both reference and experimental cell cultures. The analysis leads to the prediction that, if equal amounts of proteins of the two cultures were compared, the number of copies of a protein *p*_(i)_ per femtogram of total protein (*m*_p(av)_) would be found to differ by no more than 35%; greater differences are found for the 27 ORFs with expression ratios greater than 2.0. Similar estimates can be made for Msmeg when the appropriate value of (*n*′_aa(av)_/*n*′′_aa(av)_) is known.

#### 4.1 Cellular concentrations of DNA, ribosomes and proteins

Estimates of the cellular concentrations of DNA *et cetera* were made in order to provide further perspective for the effects of growth rate on the composition of BCG-Pasteur. The volumes (fl or μm^3^) of population-average cells were calculated from the dry cell mass on the basis of the assumptions that water accounts for 70% of the cell mass [19] and that cell density [45] is close to 1.09 fg per fl (1.09 g per ml). The results are summarized in Table 8. The composition of the slower growing cells estimated to be 11.70 fg DNA/fl (11.7 mg/ml), 14.4 fg ribosomes/fl (14.4 mg/ml) and 81.5 fg protein/fl (81.5 mg/ml). The corresponding values for faster growing cells were found to be 8.2 fg DNA/fl (8.2 mg/ml), 21.3 fg ribosomes/fl (21.3 mg/ml) and 87.3 fg protein/fl (87.3 mg/ml). In brief, the concentration of DNA decreased, the concentration of ribosomes increased by about 50% and the concentration of proteins had small change (an increase of about 7%) when the growth rate was increased threefold. Data for *E.coli* B/r are included for comparison.

**Table 8:** Concentrations of components of population-average cells of BCG-Pasteur; comparison with *E.coli* B/r.

BCG-Pasteur				
Property	Unit	Experimental culture	Reference culture	¶ *E. coli* B/r
Growth rate	h^−1^	0.01	0.03	0.42
*m*_DNA(av)_	fg	0.30	6.44	8.09
*n*_R(av)_	Ribosomes/cell	1735	3730	6800
† *m*_R(av)_	fg	7.80	16.80	30.60
*m*_p(av)_	fg	44	69	100
*m*_dc(av)_	fg	178	257	198
*m*_p(av)_/*m*_dc(av)_	fg	0.25	0.27	0.51
‡ *ν*_(av)_	fl, µm^3^	0.54	0.79	0.61
§ *c*_DNA(av)_	fg/fl, mg/ml	11.7	8.2	13.3
*c*_R(av)_	fg/fl, mg/ml	14.4	21.3	50.2
*c*_p(av)_	fg/fl, mg/ml	81.5	87.3	163.9

¶ [19].

† *m*_R(av)_, the mass of *n*_R(av)_ ribosomes was calculated on the basis of the assumption that M_r_  = 2.7×10^6^ Da so that the mass of a ribosome is 4.5×10^−3^ fg.

‡ *ν*_(av)_, the volume of a population-average cell was calculated from the equation *ν*_(av)_ fl  =  (*m*_dc(av)_/1,000)/(0.3ρ_c_) where *ρ*_c_  = 1.09 fg/fl is the density of a population-average cell [45]: *m*_dc(av)_ is thought to comprise 30% of the cell mass.

§ *c*_DNA (av)_ etc. refer to concentrations *(m*_DNA(av)_/*_V_*_(av)_) of DNA etc.

## Concluding Remarks

Both BCG-Pasteur and Msmeg were grown in carbon limited chemostats [11], [12]. Each strain was grown at the slower rate of µ =  0.01 h^−1^ and at a faster rate close to its maximum value. Growth in a chemostat has the advantage that bacteria are grown in a defined constant environment which allows the effects of growth rate to be investigated independent of other environmental parameters. Changes in the composition of the proteome with growth rate were inferred from the microarray data (see Section 3). The chemical compositions of both cultures of BCG-Pasteur were reported [21]. This additional information complements the microarray data and extends the scope of our analysis by allowing the results obtained to be related to properties of population-average cells. The data obtained for population-average cells of BCG-Pasteur are discussed in Section 4. The cellular concentrations of DNA, ribosomes and proteins were estimated and a schematic view of a cell grown at the faster rate was constructed (see Fig. 5).

Traditionally, the output from microarray studies is a series of *r*-values. For any ORF, ORF_(i)_, the *r*-value *r*_(i)_ is the ratio of the numbers of transcripts *n*_tr(i)_ per standard mass of RNA isolated from reference and experimental cultures (see equation (1) of Methods). This definition leads to *r*-values for ORFs encoding, for example, ribosomal proteins whose abundance varies with the concentration of RNA (see Section 2.4) which appear to be independent of the magnitude of the change in growth rate.

Our analysis is based on the definition that an ORF is expressed each time a copy of the encoded protein is synthesized (by translation). The translation ratio ρ_(i)_ is defined by equation (IV).
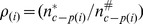
(IV)


The relation between an *r*-value r_(i)_ and ρ_(i)_ can be made apparent by substituting 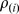 for 
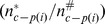
 in equations (6), (13c) and (21) (see Table 2).

Equation (21), for example, can be re-arranged to make 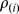 the subject (see equation (V)).
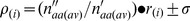
(V)


We infer (see equation (13c), Table 2) that *r*-values are equal to the ratio of the concentrations of the encoded proteins in reference and experimental cell cultures (see Section 2.2). Thus, when *r*_(i)_  = 1 the concentrations of protein *p*_(i)_ are the same in both reference and experimental cell cultures; we consider the encoded protein to be constitutively expressed. This conclusion is also evident from equation (V).

Further information was derived by expressing *r*-values as histograms (see Figs. 1 and 2). We propose that the standard deviation provides a measure of the fidelity with which the cDNA preparations reflect the distributions of the cognate mRNAs *in vivo*. Resolution of the component ORFs is increased as the standard deviation is diminished (see Figs. 2(c) and (d)). The higher standard deviation contributes to the breadth of the profile.

It was found that the profile for BCG-Pasteur, over the range *r* = 0.0–2.0 could be simulated by combining three Guassian components each centred on a particular *r*-values, each with a standard deviation of ±0.15. The major subgroup for BCG-pasteur (*r*_(i)_  = 1.0±0.15) was found to comprise 2350 of the 3475 ORFs examined. The profile for Msmeg was found to be broader; 6180 ORFs with *r*-values in the range 0.0 to 2.0 were simulated by combining five Guassian components each with a standard deviation of ±0.20. The major subgroup was found to be *r*_(i)_  = 1.0±0.20 comprising 2750 ORFs.

Bacteria readily adjust their growth rate to suit their environment. This adjustment involves changes to both the outer permeability barrier and the cytoplasmic membrane of a cell because both participate in regulating the uptake and utilization of nutrients. The results shown in Tables S1, S2, S3, S4, S5, S6, S7, S8 reveal the extent of changes in their compositions with growth rate. The greater number of two component regulatory systems and ABC transporters present in the cytoplasmic membrane of Msmeg may be factors that enable this saprophyte to grow at a faster rate, and to adapt to a wider range of conditions than is achieved by the attenuated pathogen BCG-Pasteur. The versatility of Msmeg in adapting to a changing environment is illustrated by the wide range of *r*-values found for ABC transporters.

The distinctive features of the cytoplasmic membranes of these representative members of slow and fast growing mycobacteria support the view that the two branches were established early in mycobacterial evolution [46], [47]. Slow growers include human pathogens such as the Tubercle bacillus whereas the fast growers usually live freely in the environment.

The ability to sequence the mRNA fraction of a bacterial culture has been demonstrated [43], [48], [49]. Unlike standard microarrays, this procedure is able to detect the expression of non coding RNAs, which represent unstable RNA fractions not translated into proteins (see for example [43]); which can be found either in intergenic regions or within coding regions (antisense RNA). Nowadays, the functional activities of these RNAs are becoming known (for review see [50]. Those sRNAs that relate to our analysis are newly found transcription factors, which together with conventional factors, regulate gene transcription and ensure that gene transcription and translation are coupled. These functions are in accord with the theory described in this work.

## Methods

### 1 Development of the theoretical framework

The variables considered are defined in Table 1. Empirical values of parameters obtained for microarray experiments are denoted by hash signs (reference cultures cultures) and asterisks (experimental cultures). Theoretical (authentic) values are denoted by single primes and double primes which, respectively, denote reference and experimental cultures.

Microarray analysis is a comparative method for studying gene expression. It is based on competitive hybridization of cDNA copies of samples of RNA isolated from experimental and reference cell cultures to immobilized DNA. A standard amount of RNA is used to prepare fluorescently labelled cDNA for both experimental (label *f* *) and reference (label *f* ^#^) samples.

The ratio *f* */*f* ^#^ of fluorescence immobilized to DNA representing a particular ORF (ORF_(i)_) is termed *r*_(i)_, the expression ratio. Suppose that the numbers of transcripts of ORF_(i)_ per picogram of RNA substrate used for the synthesis of experimental and reference cDNA samples are ν*_tr(i)_ and ν^#^_tr(i)_ respectively (Table 1). The expression ratio for transcripts of ORF_(i)_ is defined in equation (1)), where sigma is equal to the standard deviation of the experimental data.
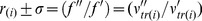
(1)


The expression ratio may be expressed in terms of properties of population-average cells, as follows. Suppose that *m*′′_RNA(av)_ and *m*′_RNA(av)_ respectively are the amounts (femtograms) of RNA per population-average cell of experimental and reference cultures then (1,000/*m*′′_RNA(av)_) and (1,000/*m*′_RNA(av)_) are the numbers of population-average cells per picogram of RNA used as substrate for cDNA synthesis for, respectively, experimental and reference samples. Hence, ν*_tr(i)_ may be expressed as the product of n*_tr(i)_, the apparent number of transcripts per population-average cell of experimental cultures and the number of population-average cells per picogram of RNA substrate as it is indicated in equation (2).
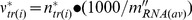
(2)


Equation (3) is appropriate for reference cultures, where *n* ^#^_tr(i)_ is, the apparent number of transcripts of ORF_(i)_, per population-average cell.
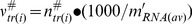
(3)


Thus, the expression ratio may be stated in terms of the properties population-average cells, as shown in equation (4).
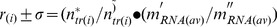
(4)


In other words the experimental and actual values of *n*_tr(i)_ are related, as shown in equation (5).
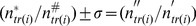
(5)


RNA is readily degraded and little is known about the efficiencies with which different RNA species are copied into cDNA and both factors are likely to affect the standard deviation. Thus, it is required to show explicitly the relation between the apparent (n*_tr(i)_, *n* ^#^_tr(i)_) and actual (*n*′′_tr(i)_, *n*′_tr(i)_) numbers of copies of transcripts of ORF_(i)_ per population-average cell. Ideally, each cDNA preparation should accurately reflect the composition of its cognate RNA. In practice, the quality of the RNA preparations are judged by the integrity of the 16S rRNA and 23S rRNA components only and not by the integrity of the mRNA fraction. However, measurements of the rates of synthesis of 16S rRNA and of ribosomal proteins such as rpsL and rplL would provide a sensitive test for the quality of mRNA [51].

As shown previously [27], when transcription and translation are coupled, *n*_c–p(i)_ the apparent gross number of copies of protein *p*_(i)_ encoded by ORF_(i)_ per population-average cell (Table 1) can be related to the expression ratio, *r*_(i)_ by means of equation (6).



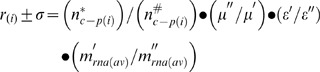
(6)


Equation (6) is based on long established principles of exponential bacterial growth namely, the concept of population-average cells [19], [52] and coupled transcription/translation [53]–[55]. These principles are defined by equations D1 to D7 presented below.

The specific protein synthesis rate *ω*_p(i)_ amino acid residues h^−1^ of protein *p*_(i)_ comprising *l*_aa(i)_ amino acids is defined by equation (D1).
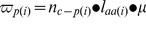
(D1)


The specific protein synthesis rate is also defined by the product of the number *n*_R(i)_ of ribosomes synthesizing *p*_(i)_ and *ε_aa(i)_* the peptide chain elongation rate of *p*_(i)_ [see equation (D2)]
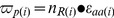
(D2)


Equation (D3) is formed by equating the right hand sides of equations (D1) and (D2) and rearranging to make *n*_R(i)_ the subject.
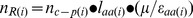
(D3)


In bacteria the processes of transcription and translation are coupled [53]–[55]; that is, transcripts are translated as they are transcribed. As a result the terms *n*_tr(i)_ and *n*_R(i)_ are related by the term *n_R(i)/tr(i)_* the number of ribosomes per nascent transcript The parameter *n*_R(i)/tr(i)_ was defined previously [27], [51]; see equation (D4).
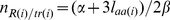
(D4)


The terms alpha (approx. 80 base-pairs) and beta (approx. 80 nucleotides) are the footprints of an initiating complex of RNA polymerase and of a ribosome respectively. Thus, *n*_R(i)_ and *n*_tr(i)_ are linked by the conversion factor *n*_R(i)/tr(i)_ (see equation (D5).
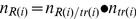
(D5)


Substitution for *n*_R(i)_ in equation (D3) and rearranging to make *n*_tr(i)_ the subject leads to equation (D6).
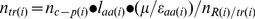
(D6)


Equation (D7) is obtained when equation (D6) is applied to reference (single prime) and experimental (double prime) cultures and *n*′′_tr(i)_ is divided by *n*′_tr(i)_. The terms *l*_aa(i)_ and *n*_R(i)/tr(i)_ cancel out.
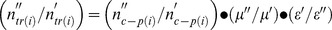
(D7)


Substitution in equation (4) of (*n* ^*^_tr(i)_/*n* ^#^_tr(i)_) for (*n*′′_tr(i)_/*n*′_tr(i)_) leads to equation (7).

 (7)

Equations (6) and (7) are equivalent. However, equation (6) is intended to make explicit the influence of experimental factors, including the fidelity of the cDNA preparations. The standard deviations of the expression ratios are not usually reported. However, we have shown (see the ‘Results and Discussion’ section) that the standard deviation calculated for the *r*-values of the 50 Zur –independent ORFs encoding ribosomal proteins provides a useful guide value which may be refined by simulation of histograms compiled from the results (see Figs. 1and 2).

#### 1.1 Genes (ORF_(k)_) encoding ribosomal proteins are a special case

Bacterial ribosomes comprise more than 50 proteins (r-proteins). With the exception of rplL each protein is present as one copy per ribosome; rplL (L7/L12) is usually present as four copies per ribosome, including two copies of L7 which is L12 acetylated at its N-terminus [56]. We suppose that, as was found for *E. coli* [20] that 98% or more of the cell's ribosomal proteins are located within ribosomes. Thus, *n*_c–p(k)_ the number of copies of a ribosomal protein per population-average cell is equal to the product of the number *n*_R(av)_ of ribosomes and the number, *n*_c–p(k)/R_, of copies of the protein per ribosome.

This property defines members of the ORF_(k)_ group of encoded proteins (see Results and Discusion). Thus, the ratio *n*′′_c–p(k)/_ *n*′_c–p(k)_ is equal to the ratio *n*′′_R(av)_/*n*′_R(av)_. In turn, *n*_R(av)_ and *m*_RNA(av)_ are related. In exponentially growing cells of *E. coli* the composition of *m*_RNA(av)_ is reported to be 83% rRNA, 16% tRNA and 1% mRNA [19]. In principle, *n*_R(av)­_ can be calculated from *m*_RNA(av)_; suppose that *n*_R(av)_  =  *φ* • *m*_RNA(av)_, where *φ* is a constant. Hence, the following equalities apply [see equation (8)]. The constant *φ* cancels out.
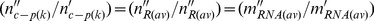
(8)


Hence, for the family of 50 Zur independent ORFs encoding ribosomal proteins (ORF_(k)_) equation (7) reduces to equation (9) where <*r*_(k)_> is the average value of *r*_(k)_.

 (9)

Two examples illustrate the significance of equation (9). First, as shown by equation (10), *ε*′_aa(k)_, can be expressed in terms of *ε*′′_aa(k)_ since µ′ and µ′′ are known and *r*_(k)_ can be measured.
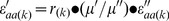
(10)


Secondly, when µ′ > µ′′ then *ε*′_aa(k)_ > *ε*′′_aa(k)_, and the empirical plot of µ′/µ′′ versus *ε*′_aa(k)_/*ε* ′′_aa(k)_ yields equation (11a), as shown by Fig. 3.
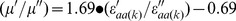
(11a)


The term 1.69 is the reciprocal of the limiting value of <*r*_(k)_>  = 0.59 found when µ′ >> µ′′. Rearrangement leads to equation (11b) with *ε*′_aa(k)_/*ε*′′_aa(k)_ as the subject.
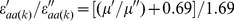
(11b)


When µ′ < µ′′ the plot is non-linear and then equations (11a) and (11b) no longer apply.

#### 1.2 A practical form of the general equation

Although *ε*_aa(i)_ may depend on properties of the particular ORF_(i)_ the ratio (*ε*′_aa(i)_/*ε*′′_aa(i)_) is likely to be a constant which is dependent on the ratio µ′/µ′′ of the growth rates. On the basis of this assumption equation (6) may be written as equation (12) by substituting *r*_(k)_ for (µ′′/µ′)•(*ε*'_aa(i)_/*ε*′′_aa(i)_).
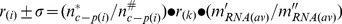
(12)


Thus equation (12) shows that *r*_(i)_ is directly proportional to (n*_c–p(i)_/*n* ^#^_c–p(i)_). Re-arrangement of equation (12) leads to equations (13a) and (13b).
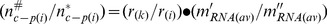
(13a)

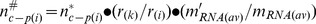
(13b)


Equation (13c) is the practical form of equation (13b).
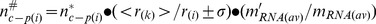
(13c)


The ratio (n*_c–p(i)_/*n* ^#^_c–p(i)_) may be evaluated when <*r*_(k)_>, *m*′_RNA(av)_ and *m*′′_RNA(av)_ are known. The latter three parameters are constants for a particular microarray. Hence, the expression ratio is directly proportional to (n*_c–p(i)_/*n* ^#^_c–p(i)_).

#### 1.3 Quantification of microarray data

Knowledge of the macromolecular compositions of the cultures compared in microarray experiments is needed to allow expression ratios to be expressed in terms of *n* ^#^_c–p(i)_ and n*_c–p(i)_ (see equation (12) for example). The properties of population-average cells usually reported include dry cell mass (*m*_dc(av))_, protein content (*m*_p(av)_ or *n*_aa(av))_ and RNA content (*m*_RNA(av)_).

The specific protein synthesis rate (*ω*_p(av)_ or *ω*_aa(av)_) for exponentially growing cells is given by equation (14).
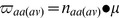
(14)


Equation (15) is an alternative form of equation (14) which includes, *ε*_aa(av)_, the peptide chain elongation rate. The term *β*_R_ is the fraction of ribosomes actively synthesizing protein and *n*_R(av)_ is the number of ribosomes per population-average cell.
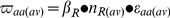
(15)


Equating the right hand sides of equations (14) and (15) and rearranging to make *n*_aa(av)_ the subject leads to equation (16).
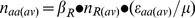
(16)


Equations (17) and (18) are forms of equation (16) specifying reference (single prime) and experimental (double prime) cultures.
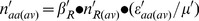
(17)

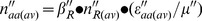
(18)


Division of equation (17) by equation (18) leads to equation (19).
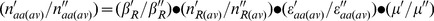
(19)


Equation (19) reduces to equation (20) on the basis of the following considerations; *β*′≈*β*′′, *n*′_R(av)_/*n*′′_R(av)_  =  *m*′_RNA(av)_/*m*′′_RNA(av_ and (µ′′/µ′) • (*ε*′_aa(av)_/*ε*′′_aa(av)_)  =  *r*_(k)_ ≈ <*r*_(k)_>.
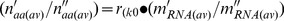
(20)


Thus, equation (20) defines *r*_(k)_ as the ratio of RNA (or the number of ribosomes) to protein in experimental cultures divided by the RNA (or the number of ribosomes) to protein ratio in reference cultures.

Equation (12) can be simplified [see equation (21)] by substituting (*n*′_aa(av)_/*n*′′_aa(av)_) for *r*(_k)_ • (*m*′_RNA(av)_/*m*′′_RNA(av)_).
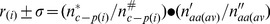
(21)


Equation (21) is alternative form of equation (4); the ratios (*m*′_RNA(av)_/*m*′′_RNA(av)_) and (*n*′_aa(av)_/*n*′′_aa(av)_) are related through *r(*_k)_, as shown in equation (20).

#### 1.4 The significance of r-values of unity

It is convenient to refer to ORFs that have expression ratios of unity as members of the subgroup ORF_(j)_. The numbers of copies of the encoded protein, *p*_(j)_ per population-average cell is then directly proportional to cell size; and the cellular concentration of *p*_(j)_ is independent of the specific growth rate. Suppose that the ratio of the numbers of cells used in the preparation of reference and experimental cDNA samples are *n*′_cells_ and *n*′′_cells_ respectively. Equation (22) then applies to the subgroup ORF_(j)_.
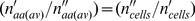
(22)


Equation (23a) is derived by substituting for (*n*′_aa(av)_/*n*′′_aa(av)_) in equation (21) when *r*  =  *r*_(j)_  = 1 and then rearranging
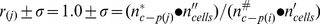
(23a)


or
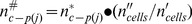
(23b)


Similarly, when *r*_(i)_ <1 equation (24) applies which reveals that there is higher concentration of *p*_(i)_ in the reference culture compared with the experimental culture.
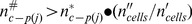
(24)


Conversely, when *r*_(i)_ >1 equation (25) applies which reveals that there is higher concentration of protein *p*_(i)_ in the experimental culture compared with the reference culture
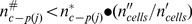
(25)


In brief, *r*_(i)_ is a measure of the relative concentrations of the encoded protein, *p*_(i)_ in cultures of reference and experimental cells, as shown in equation (21).

We considered that errors in expression ratios resulted from many small errors, including deficiencies in the quality of cDNA preparations. The Central Limit Theory was applied on the basis of this assumption and the errors were considered to have a normal (Gaussian) distribution: (http://introcs.cs.princeton.edu/java/11gaussian).

### 2 Changes in the protein moiety of BCG-Pasteur with growth rate inferred from microarray data

The equations summarized in Table 2 relate the number of transcripts of ORF_(i)_ with the gross number of copies of the encoded protein *p*_(i)_ ; neither the secretion nor degradation of a protein are taken into account by our analysis. However it is assumed that the majority of proteins are stable so that the gross protein content is sum *n*_aa(av)_ + δ *n*_aa(av)_ ; where *n*_aa(av)_ refers to stable proteins and *δ n*_aa(av)_ refers to secreted and degraded proteins. It is assumed that *δ n*_aa(av)_ is very small compared with *n*_aa(av)_. Differences in the protein moieties of reference and experimental cultures were identified by considering the component families of ORFs ORF_(j)_, ORF_(k)_ *et cetera* deduced from the simulation studies (see Fig. 2).

The 3475 ORFs of BCG-Pasteur investigated were expressed in both reference and experimental cultures.

The protein composition (see the legend to Fig. 2a) of the reference culture is given by equation (R1); the 27 ORFs with *r*-values greater than 2.0 comprise the subgroup alpha.
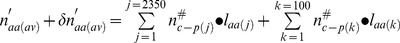
(R1)

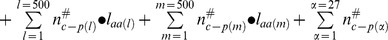



However, *n* ^*^_c–p(j)_ *et cetera* are related to *n* ^#^_c–p(j)_ *et cetera* by equation (21b) which is a rearrangement of equation (21).
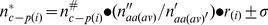
(21b)


Substitution for *n* ^#^_c–p(i)_ *et cetera* and for (*n*′′_aa(av)_/*n*′_aa(av)_)  = 1.56 leads to equation (R2).
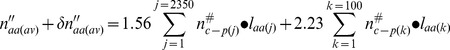



 (R2)

When equal amounts of proteins of the two cultures are compared, the concentration (number of copies per femtogram of a protein) of the *p*_(j)_ subgroup is the same in both cultures; the concentrations of a protein *p*_(k)_ and *p*_(l)_ are diminished in the experimental culture by 30% and 20% respectively; the concentration of a protein of the *p*_(m)_ subgroup is increased in the experimental culture by 35%. Similar estimates can be made for Msmeg when the appropriate value of (*n*′_aa(av)_/*n*′′_aa(av)_) is known.

## Supporting Information

Material S1 Supporting data for equations 11a and b.
(DOC)

Material S2 Supporting data for Table 4.
(DOC)

Table S1 Effect of growth conditions on the expressions of genes of the Zur regulon of BCG-Pasteur.
(DOC)

Table S2 Effects of growth rate on the expression of genes of the toxin/antitoxin systems of BCG-Pasteur and Msmeg.
(DOC)

Table S3 Effects of growth rate on the expression of porin genes of Msmeg.
(DOC)

Table S4 Effects of growth rate on the expression of genes of the two component systems of BCG-Pasteur.
(DOC)

Table S5 Effects of growth rate on the expression of genes of the two component systems of Msmeg with orthologues found in other fast grower mycobacteria.
(DOC)

Table S6 Effects of growth rate on the expression of genes of the ABC transporters of BCG-Pasteur.
(DOC)

Table S7 Effects of growth rate on the expression of genes of Msmeg encoding components of ABC transporters without orthologues found in BCG-Pasteur.
(DOC)

Table S8 Effects of growth rate on the expression of genes of serine/threonine protein kinases.
(DOC)
