# Supplementary material for: Adaptation of Mycobacteria to Growth Conditions: A Theoretical Analysis of Changes in Gene Expression Revealed by Microarrays
Source: PLoS One. 2013 Apr 12;8(4):e59883. doi: 10.1371/journal.pone.0059883 (PMC3625197; doi:10.1371/journal.pone.0059883)
Supplement: Material S1 — Supporting data for equations 11a and b. (DOC) [file pone.0059883.s001.doc]

**Supporting data for Equations (11a and b)**

We considered the hypothesis that equation (11a) (Table 2) applies to all exponentially growing bacteria when the specific growth rate exceeds the time needed to replicate the genome so that, at least to a first approximation, the mass of DNA per cell is independent of growth rate. This possibility was found to be supported by the available data for *Escherichia coli* B/r [19]. Data for growth rates of µ = 0.42 h^-1^(*ε*"_aa(av)_ = 43,200 amino acid residues h^-1^ ) and µ = 0.69 h^-1^ (*ε*'_aa(av)_ = 57,600 amino acid residues h^-1^) The numerical value of the ratio *ε*'_aa(av)_/*ε*"_aa(av)_ is 1.33 compared with the value of 1.38 calculated from (µ'/µ") by applying equation (11b), see Table 2. This correlation between reported and theoretical values supports our hypothesis. Please note that our analysis does not apply to faster growing cultures of *E. coli* when the time needed to replicate the genome exceeds the specific growth rate; then, the mass of DNA per cell depends on growth rate and new born cells are merodiploid [19].
