# Supplementary material for: Adaptation of Mycobacteria to Growth Conditions: A Theoretical Analysis of Changes in Gene Expression Revealed by Microarrays
Source: PLoS One. 2013 Apr 12;8(4):e59883. doi: 10.1371/journal.pone.0059883 (PMC3625197; doi:10.1371/journal.pone.0059883)
Supplement: Material S2 — Supporting data for Table 4. (DOC) [file pone.0059883.s002.doc]

**Supporting data for Table 4**

*Estimation of the number of genome equivalents per population-average cell*. The ratios DNA : RNA : protein are usually obtained by chemical analysis of cell lysates. Additional information is needed to convert these ratios to properties of the population-average cell. It is essential to know the value of the number of genome equivalents per population-average cell. The information needed is related to the duration and timing of DNA synthesis during the cell division cycle. The relevant periods are the interval (B hours) between cell division and the start of DNA synthesis, the period (C hours) when DNA is replicated and the period (D hours) between the completion of DNA synthesis and cell division. In mycobacteria the processes of DNA replication, chromosome segregation and septum formation are linked [57]. Except for measurements of the C-periods in *M. smegmatis* and *M. tuberculosis* [58] there are no data for the B, C and D periods of mycobacterial species. However, knowledge of the C and D periods are needed to evaluate the number *n*_g_ of genome equivalents per population-average cell. Estimates of the C and D periods were obtained in the following way. The duration of the C period was obtained by means of equation (S1) where *l*_g_ base-pairs and 2*ε*_DNA(av)_ base-pairs h^-1^ represent the rate of bi-directional DNA synthesis starting at the origin of replication.

(S1)

*ε*_DNA(av)_ was obtained from an empirical equation (S2) based on three known C periods where µ is the specific growth rate.

(S2)

The D period was calculated from equation (S3) where *t*_D_ is the generation time.

(S3)

Implicit in equation (S3) is the notion that for slow growing bacteria D < B. Values of *n*_g(av)_ were then calculated from equation (S4), see [59].

(S4)

The values of *n*_g(av)_ calculated for *M. bovis* BCG were very similar for the two growth rates of interest.

| **Supporting data for Table 4.** Comparisons of observed and calculated values of the C period of reference strains and values calculated for BCG-Pasteur. | | | | | | |
| --- | --- | --- | --- | --- | --- | --- |
| Bacterial strain | Genome size  (Mega base pairs) |  | 2*ε*_DNA(av)_ base pairs h^-1^ | | C period (h) | |
|  |  | *t*_D_ | Calculated^†^ | Observed | Calculated^†^ | Observed |
|  |  |  |  |  |  |  |
| *E. coli* B/r^#^ | 4.64 | 1.67 | 5.59 x 10^6^ | 3.93 x 10^6^ | 0.83 | 1.12 |
| *M. smegmatis*^‡^ | 7.00 | 3.00 | 4.00 x 10^6^ | 4.00 x 10^6^ | 1.75 | 1.75 |
| *M. tuberculosis*^‡^ | 4.40 | 24.00 | 0.42 x 10^6^ | 0.43 x 10^6^ | 10.48 | 10.33 |
| *M. bovis* BCG-Pasteur | 4.40 | 23.00 | 0.43 x 10^6^ | na | 10.26 | na |
| *M. bovis* BCG-Pasteur | 4.40 | 69.00 | 0.42 x 10^6^ | na | 22.13 | na |
|  |  |  |  |  |  |  |

*t*_D_, generation time (h): *ε*_DNA(av)_ base-pairs h^-1^ is the rate of DNA synthesis per replication fork: 2*ε*_DNA(av)_, the rate of DNA synthesis for bi-directional replication. C period, the period of DNA synthesis; na, no results available.

^#^, [19].

^‡^, [58]. ^†^, calculated by means of equations S1 and S2.

**References**

1. Bremer H, Dennis PP (1996) Modulation of chemical composition and other parameters of the cell growth rate In *Escherichia coli* an*d* *Salmonella:* Cellular and Molecular Biology, 2nd edn. pp. 1553-1568. Edited by F. C. Neidhardt and others. Washington DC: ASM Press.
2. Klann AG, Belanger AE, Abanes-De Mello A, Lee JY, Hatfull GF (1998) Characterization of the *dnaG* locus in *Mycobacterium smegmatis* reveals linkage of DNA replication and cell division. J Bacteriol. 180: 65-72.
3. Hiriyanna KT, Ramakrishnan T (1986) Deoxyribonucleic acid replication time in *Mycobacterium tuberculosis* H37 Rv. Archives of microbiology 144: 105-109.
4. Cooper S, Helmstetter CE (1968) Chromosome replication and the division cycle of *Escherichia coli* B/r. J Mol Biol. 31: 519-540.
