# Supplementary material for: Adaptation of Mycobacteria to Growth Conditions: A Theoretical Analysis of Changes in Gene Expression Revealed by Microarrays
Source: PLoS One. 2013 Apr 12;8(4):e59883. doi: 10.1371/journal.pone.0059883 (PMC3625197; doi:10.1371/journal.pone.0059883)
Supplement: Table S1 — Effect of growth conditions on the expressions of genes of the Zur regulon of BCG-Pasteur. (DOC) [file pone.0059883.s003.doc]

| **Table S1.** Effect of growth conditions on the expressions of genes of the Zur regulon of BCG-Pasteur [18]. | | | |
| --- | --- | --- | --- |
|  |  | *r*-value | |
| Locus tag | Gene | Effect of growth rate | Zinc deficiency |
|  |  | | |
| BCG_0138c (Rv0105c) | *rpmB1* | 2.48 | 2.7 |
| BCG_0139 (Rv0106) |  | 7.03 | 8.1 |
| BCG_0269 (Rv0232) |  | 1.00 | 1.9 |
| BCG_0320 (Rv0280) | *ppe3* | 6.47 | 9.6 |
| BCG_0321 (Rv0281) |  | 1.32 | 3.6 |
| BCG_0322 (Rv0282) |  | 2.11 | ND |
| BCG_0323 (Rv0283) |  | 2.02 | 4.3 |
| BCG_0324 (Rv0284) |  | 1.83 | 3.0 |
| BCG_0325 (Rv0285) | *pe5* | nr | 3.4 |
| BCG_0326 (Rv0286) | *ppe4* | 1.73 | 3.3 |
| BCG_0327 (Rv0287) | *esxG* | 1.88 | 3.0 |
| BCG_0328 (Rv0288) | *esxH* | 1.85 | 3.2 |
| BCG_0329 (Rv0289) |  | 2.18 | 3.2 |
| BCG_0330 (Rv0290) |  | 2.08 | 3.7 |
| BCG_0331 (Rv0291) | *mycP3* | 1.84 | 3.1 |
| BCG_0332 (Rv0292) |  | 2.06 | 3.3 |
| BCG_1255 (Rv1195) | *pe11* | nr | 2.4 |
| BCG_1893 (Rv1857) | *modA* | 0.73 | 1.9 |
| BCG_1906c (Rv1870c) |  | 0.82 | 2.1 |
| BCG_2074c (Rv2055c) | *rpsR2* | 2.58 | 12.8 |
| BCG_2075c (Rv2056c) | *rpsN2* | 6.02 | 22.5 |
| BCG_2076c (Rv2057c) | *rpmG1* | 4.81 | 23.3 |
| BCG_2077c (Rv2058c) | *rpmB2* | 7.69 | 30.1 |
| BCG_2078 (Rv2059) | *Zur* | 2.58 | 5.8 |
| BCG_2079 (Rv2060) |  | 0.74 | 6.3 |
| BCG_3011c (Rv2990c) |  | 2.96 | 4.1 |
| BCG_3039c (Rv3017c) | *esxQ* | 1.08 | 2.1 |
| BCG_3042c (Rv3019c) | *esxR* | 1.43 | 3.9 |
| BCG_3043c (Rv3020c) | *esxS* | 1.39 | 2.5 |
| BCG_3044c (Rv3022c) | *ppe47* | 1.36 | 2.2 |
| BCG_3352c (Rv3229c) |  | 1.95 | 2.9 |
| BCG_3676c (Rv3612c) |  | 0.86 | 2.1 |
|  |  |  |  |

nr, no results. Corresponding loci in *M. tuberculosis* H37Rv, according to [11], are indicated between brackets.
