# Supplementary material for: Adaptation of Mycobacteria to Growth Conditions: A Theoretical Analysis of Changes in Gene Expression Revealed by Microarrays
Source: PLoS One. 2013 Apr 12;8(4):e59883. doi: 10.1371/journal.pone.0059883 (PMC3625197; doi:10.1371/journal.pone.0059883)
Supplement: Table S2 — Effects of growth rate on the expression of genes of the toxin/antitoxin systems of BCG-Pasteur and Msmeg. (DOC) [file pone.0059883.s004.doc]

| **Table S2.** Effects of growth rate on the expression of genes of the toxin /antitoxin systems of BCG-Pasteur and Msmeg. | | | | | |
| --- | --- | --- | --- | --- | --- |
| Gene | BCG-Pasteur | | Gene | Msmeg | |
|  | Locus tag | *r*-value |  | Locus tag | *r*-value |
|  |  |  | |  |  |
| vapBC1 | BCG_0096 (Rv0065) | 0.82 | phd | MSMEG_1277 | 0.9 |
| vapBC-2 | BCG_0340 (Rv0300) | 0.81 | doc | MSMEG_1278 | 0.71 |
| vapBC-3 | BCG_0594c (Rv0549c) | 1.14 | vapB | MSMEG_1283 | 1.15 |
| vapBC-3 | BCG_0595c (Rv0550c) | 1.03 | vapC | MSMEG_1284 | 1.16 |
| vapBC-4 | BCG_0641c (Rv0595c) | 0.92 | mazE | MSMEG_4447 | 1.03 |
| vapBC-5 | BCG_0672 (Rv0626) | 1.03 | mazF | MSMEG_4448 | 0.66 |
| vapBC5 | BCG_0673 (Rv0627) | 0.89 |  |  |  |
| mazEF-2 | BCG_0708c (Rv0659c) | 0.9 |  |  |  |
| mazEF-2 | BCG_0709c (Rv0660c) | 0.9 |  |  |  |
| vapBC-7 | BCG_0710c (Rv0661c) | 0.97 |  |  |  |
| vapBC-7 | BCG_0711c (Rv0662c) | 0.82 |  |  |  |
| vapBC-8 | BCG_0713 (Rv0664) | 1.02 |  |  |  |
| vapBC-8 | BCG_0714 (Rv0665) | 0.9 |  |  |  |
| vapBC-9 | BCG_1014 (Rv0960) | 0.84 |  |  |  |
| mazEF-3 | BCG_1162c (Rv1102c) | 0.75 |  |  |  |
| relBE-1 | BCG_1306c (Rv1246c) | 0.84 |  |  |  |
| relBE-1 | BCG_1307c (Rv1247c) | 0.8 |  |  |  |
| vapBC-10 | BCG_1458c (Rv1397c) | 0.93 |  |  |  |
| vapBC-10 | BCG_1459c (Rv1398c) | 0.79 |  |  |  |
| mazEF-4 | BCG_1557 (Rv1494) | 0.76 |  |  |  |
| mazEF-4 | BCG_1558 (Rv1495) | 0.87 |  |  |  |
| vapBC-11 | BCG_1612 (Rv1560) | 0.71 |  |  |  |
| vapBC-11 | BCG_1613 (Rv1561) | 0.88 |  |  |  |
| vapBC-12 | BCG_1759c (Rv1720c) | 0.94 |  |  |  |
| vapBC-13 | BCG_1873c (Rv1838c) | 0.76 |  |  |  |
| vapBC-13 | BCG_1874c (Rv1839c) | 0.85 |  |  |  |
| mazEF-5 | BCG_1981c (Rv1942c) | 1.12 |  |  |  |
| mazEF-5 | BCG_1982c (Rv1943c) | 0.93 |  |  |  |
| vapBC-14 | BCG_1992 (Rv1953) | 0.91 |  |  |  |
| vapBC-14 | BCG_1991 (Rv1952) | 0.79 |  |  |  |
| higBA-1 | BCG_1995 (Rv1956) | 0.85 |  |  |  |
| mazEF-6 | BCG_2008c (Rv1991c) | 1.03 |  |  |  |
| vapBC-15 | BCG_2026 (Rv2009) | 0.71 |  |  |  |
| vapBC-15 | BCG_2027 (Rv2010) | 0.97 |  |  |  |
| parDE-2 | BCG_2159c (Rv2142c) | 1.11 |  |  |  |
| mazEF-8 | BCG_2291c (Rv2274c) | 0.84 |  |  |  |
| vapBC-18 | BCG_2568 (Rv2545) | 1.04 |  |  |  |
| vapBC-19 | BCG_2571 (Rv2548) | 1.07 |  |  |  |
| vapBC-20 | BCG_2573c (Rv2550c) | 0.67 |  |  |  |
| vapBC-21 | BCG_2773c (Rv2757c) | 1.03 |  |  |  |
| vapBC-21 | BCG_2601c (Rv2578c) | 0.79 |  |  |  |
| vapBC-22 | BCG_2849c (Rv2829c) | 0.91 |  |  |  |
| vapBC-23 | BCG_2885 (Rv2863) | 1.01 |  |  |  |
| relBE-3 | BCG_3430 (Rv3358) | 0.89 |  | | |
|  |  |  | |  |  |

The corresponding loci in *M. tuberculosis* H37Rv, according to [11], are indicated between brackets in BCG-Pasteur .
