# Supplementary material for: Adaptation of Mycobacteria to Growth Conditions: A Theoretical Analysis of Changes in Gene Expression Revealed by Microarrays
Source: PLoS One. 2013 Apr 12;8(4):e59883. doi: 10.1371/journal.pone.0059883 (PMC3625197; doi:10.1371/journal.pone.0059883)
Supplement: Table S3 — Effects of growth rate on the expression of porin genes of Msmeg. (DOC) [file pone.0059883.s005.doc]

| **Table S3.** Effects of growth rate on the expression of porin genes of Msmeg. | | |
| --- | --- | --- |
| Locus Tag | Gene | *r*-value |
|  |  |  |
| MSMEG_0520 | (*mspB*) | 1.78 |
| MSMEG_0965 | (*mspA*) | 0.61 |
| MSMEG_5483 | (*mspC*) | 2.04 |
| MSMEG_6466 | (*mspD*) | 1.62 |
|  |  |  |
