# Supplementary material for: Adaptation of Mycobacteria to Growth Conditions: A Theoretical Analysis of Changes in Gene Expression Revealed by Microarrays
Source: PLoS One. 2013 Apr 12;8(4):e59883. doi: 10.1371/journal.pone.0059883 (PMC3625197; doi:10.1371/journal.pone.0059883)
Supplement: Table S4 — Effects of growth rate on the expression of genes of the two component systems of BCG-Pasteur. (DOC) [file pone.0059883.s006.doc]

| **Table S4.** Effects of growth rate on the expression of genes of the two component systems of BCG-Pasteur. | | | | | |
| --- | --- | --- | --- | --- | --- |
| (a) Genes with orthologues found in Msmeg. | | | | | |
|  | | BCG-Pasteur | | Msmeg | |
| Gene | Function | Locus tag | *r*-value | Locus tag | *r*-value |
|  |  |  |  |  |  |
| *sen*X3 | S | BCG_0531 (Rv0490) | 1.24 | MSMEG_0936 | 1.50 |
| *reg*X3 | R | BCG_0532 (Rv0491) | 0.91 | MSMEG_0937 | 1.41 |
| *pho*P | S | BCG_0809 (Rv0757) | 0.94 | MSMEG_5872 | 2.38 |
| *pho*R | R | BCG_0810 (Rv0758) | 0.98 | MSMEG_5870 | 0.73 |
| *prr*A | S | BCG_0955c (Rv0903c) | 1.07 | MSMEG_0244 | 0.71 |
| *prr*B | R | BCG_0954c (Rv0902c) | 1.17 | MSMEG_0246 | 0.36 |
| *prr*B* | R | Not applicable |  | MSMEG_2793 | 0.43 |
| *prr*B* | R | Not applicable |  | MSMEG_5663 | 0.74 |
| *mpr*A | S | BCG_1036 (Rv0981) | 0.87 | MSMEG_5488 | 1.47 |
| *mpr*B | R | BCG_1037 (Rv0982) | 1.20 | MSMEG_5487 | 1.20 |
| *kdp*D | S | BCG_1085c (Rv1028c) | 1.20 | MSMEG_5372 | 1.89 |
| *kdp*D* | S | Not applicable |  | MSMEG_5395 | 1.87 |
| *kdp*E | R | BCG_1084c (Rv1027c) | 1.16 | MSMEG_5396 | 1.21 |
| *trc*S | S | BCG_1090c (Rv1032c) | 0.69 | MSMEG_2915 | 1.81 |
| *trc*R | R | BCG_1091c (Rv1033c) | 0.68 | MSMEG_2916 | 3.56 |
| *dev*S | S | BCG_3155c (Rv3132c) | 1.04 | MSMEG_5241 | 1.49 |
| *dev*S* | S | BCG_2046c (Rv2027c) | 1.05 | Not applicable |  |
| *dev*R | R | BCG_3156c (Rv3133c) | 1.39 | MSMEG_5244 | 1.95 |
| *devR** | R | Not applicable |  | MSMEG_3944 | 1.04 |
| *mtr*B | S | BCG_3274c (Rv3245c) | 0.96 | MSMEG_1875 | 1.47 |
| *mtr*A | R | BCG_3275c (Rv3246c) | nr | MSMEG_1874 | 0.98 |
|  | oR | BCG_0298c (Rv0260c) | 1.33 | MSMEG_0432 | 0.37 |
|  | oS | BCG_3340c (Rv3220c) | 1.44 | MSMEG_1918 | 0.65 |
|  |  |  |  |  |  |

R, regulator; S, Sensor; o, orphan or unpaired component; *, homologous component;

nr, no result.

The corresponding loci in *M. tuberculosis* H37Rv, according to [11], are indicated between brackets in BCG-Pasteur .

The two component system *dev*R/S is also known as *dos*R/S in *M. tuberculosis*. *dev*S*, the homologous component is also known as *dos*T in *M. tuberculosis*.

| **Table S4 (cont).** Effects of growth rate on the expression of genes of the two component systems of BCG-Pasteur. | | | |
| --- | --- | --- | --- |
| (b) Genes without orthologues found in Msmeg. | | | |
| Gene | Function | Locus tag | *r*-value |
|  |  |  |  |
| *tcr*B2 | R | BCG_0646c (Rv0600c) | 1.71 |
| *tcr*B1 | S | BCG_0647c (Rv0601c) | 1.29 |
| *tcr*A | S | BCG_0648c (Rv0602c) | nr |
| *nar*L | R | BCG_0896c (Rv0844c) | 0.92 |
| *nar*S | S | BCG_0897 (Rv0845) | 1.24 |
| *tcr*Y | S | BCG_3823c (Rv3764c) | 1.16 |
| *tcr*X | R | BCG_3824c (Rv3765c) | 1.53 |
|  | oR | BCG_0870 (Rv0818) | nr |
|  | oR | BCG_ 1664 (Rv1626) | 0.67 |
|  | oR | BCG_2905 (Rv2884) | 1.41 |
|  | oR | BCG_3166 (Rv3143) | 0.93 |
|  |  |  |  |

R, regulator; S, Sensor; o, orphan or unpaired component; *, homologous component; nr, no result.

The corresponding loci in *M. tuberculosis* H37Rv, according to [11], are indicated between brackets.
