# Supplementary material for: Adaptation of Mycobacteria to Growth Conditions: A Theoretical Analysis of Changes in Gene Expression Revealed by Microarrays
Source: PLoS One. 2013 Apr 12;8(4):e59883. doi: 10.1371/journal.pone.0059883 (PMC3625197; doi:10.1371/journal.pone.0059883)
Supplement: Table S5 — Effects of growth rate on the expression of genes of the two component systems of Msmeg with orthologues found in other fast grower mycobacteria. (DOC) [file pone.0059883.s007.doc]

| **Table S5.** Effects of growth rate on the expression of genes of the two component systems of Msmeg with orthologues found in other fast grower mycobacteria. | | |
| --- | --- | --- |
| Function | Locus tag | *r*-value |
|  |  |  |
| S | MSMEG_0458 | 1.18 |
| R | MSMEG_0459 | 1.14 |
| R | MSMEG_0981 | 1.80 |
| R | MSMEG_0983 | 1.32 |
| S | MSMEG_2248 | 1.15 |
| R | MSMEG_2251 | 1.88 |
| S | MSMEG_2804 | 0.87 |
| R | MSMEG_2806 | 1.01 |
| R | MSMEG_2807 | 0.97 |
| R | MSMEG_3447 | 1.76 |
| S | MSMEG_3448 | 1.19 |
| S | MSMEG_4968 | 3.01 |
| R | MSMEG_4969 | 2.26 |
| R | MSMEG_6236 | 2.41 |
| S | MSMEG_6238 | 3.07 |
| oS | MSMEG_0854 | 3.82 |
| oR | MSMEG_2064 | 3.97 |
| oS | MSMEG_3239 | 1.26 |
| oR | MSMEG_4211 | 6.89 |
| oR | MSMEG_4378 | 1.16 |
| oR | MSMEG_5306 | 1.53 |
| oR | MSMEG_6128 | 0.93 |
|  |  |  |

Genes of Msmeg with orthologues usually found in other fast growers but absent from slow growers, including BCG-Pasteur.
