# Supplementary material for: Adaptation of Mycobacteria to Growth Conditions: A Theoretical Analysis of Changes in Gene Expression Revealed by Microarrays
Source: PLoS One. 2013 Apr 12;8(4):e59883. doi: 10.1371/journal.pone.0059883 (PMC3625197; doi:10.1371/journal.pone.0059883)
Supplement: Table S6 — Effects of growth rate on the expression of genes of the ABC transporters of BCG-Pasteur. (DOC) [file pone.0059883.s008.doc]

| **Table S6.** Effects of growth rate on the expression of genes of the ABC transporters of BCG-Pasteur. | | | | |
| --- | --- | --- | --- | --- |
| (a) Genes with orthologues found in M. smeg | | | | |
|  | BCG-Pasteur | | M. smeg | |
| Gene | Locus tag | *r*-value | Locus tag | *r*-value |
|  |  |  | |  |
|  | BCG_0231 (Rv0194) | 1.18 | MSMEG_5659 | 0.81 |
|  | BCG_0303c (Rv0265c) | 1.18 | MSMEG _0438 | 0.80 |
| *glnH* | BCG_0450c (Rv0411c) | 1.17 | MSMEG_0787 | !.57 |
| *pitA* | BCG_0589c (Rv0545c) | 1.09 | MSMEG _1064 | 1.17 |
| *mkl* | BCG_0704 (Rv0655) | 0.83 | MSMEG _1366 | 1.38 |
| *phoT* | BCG_0872 (Rv0820) | 1.00 | MSMEG_5779 | 2.08 |
| *pstS3* | BCG_0980 (Rv0928) | 0.64 | MSMEG_5782 | 0.90 |
| *pstC2* | BCG_0981 (Rv0929) | 0.91 | MSMEG_5781 | 1.25 |
| *pstA1* | BCG_0982 (Rv0930) | 0.67 | MSMEG_5780 | 1.17 |
|  | BCG_1041 (Rv0986) | 0.88 | MSMEG_2524 | 0.14 |
| *pstB* | BCG_0987 (Rv0933) | 0.67 | MSMEG _5779 | 2.08 |
| *pstS1* | BCG_0988 (Rv0934) | 0.70 | MSMEG _5782 | 0.90 |
| *pstC1* | BCG_0989 (Rv0935) | 1.27 | MSMEG _5781 | 1.25 |
| *pstA2* | BCG_0990 (Rv0936) | 1.09 | MSMEG _5780 | 1.17 |
|  | BCG_1277c (Rv1217c) | 1.16 | MSMEG _5076 | 1.12 |
|  | BCG_1278c (Rv1218c) | 0.75 | MSMEG _5075 | 1.18 |
| *lpqY* | BCG_1295 (Rv1235) | 1.10 | MSMEG _5061 | 2.06 |
| *sugA* | BCG_1296 (Rv1236) | 0.99 | MSMEG _5060 | 1.40 |
| *sugB* | BCG_1297 (Rv1237) | 0.68 | MSMEG _5059 | 1.20 |
| *sugC* | BCG_1298 (Rv1238) | 0.84 | MSMEG _5058 | 1.37 |
|  | BCG_1331c (Rv1272c) | 1.12 | MSMEG_5009 | 0.72 |
|  | BCG_1332c (Rv1273c) | 1.17 | MSMEG_5008 | 0.80 |
| *oppA* | BCG_1339c (Rv1280c) | 0.96 | MSMEG _4999 | 1.03 |
| *oppD* | BCG_1340c (Rv1281c) | 1.10 | MSMEG _4997 | 0.59 |
| *oppC* | BCG_1341c (Rv1282c) | 0.83 | MSMEG _4996 | 0.44 |
| *oppB* | BCG_1342c (Rv1283c) | 1.21 | MSMEG _4995 | 0.76 |
|  | BCG_1410 (Rv1348) | 1.91 | MSMEG _6554 | 0.70 |
|  | BCG_1411 (Rv1349) | 1.83 | MSMEG _6553 | 0.56 |
|  | BCG_1517c (Rv1456c) | 1.04 | MSMEG _3117 | 0.33 |
|  | BCG_1518c (Rv1457c) | 0.95 | MSMEG _3118 | 0.59 |
|  | BCG_1519c (Rv1458c) | 1.08 | MSMEG _3119 | 0.50 |
|  | BCG_1524 (Rv1463) | 0.93 | MSMEG _3124 | 1.03 |
|  | BCG_1534 (Rv1473) | 1.33 | MSMEG _3140 | 0.56 |
| *cydD* | BCG_1659c (Rv1621c) | 1.19 | MSMEG_3231 | 0.52 |
| *cydB* | BCG_1660c (Rv1622c) | 1.47 | MSMEG_3232 | 0.23 |
|  | BCG_1724c (Rv1686c) | 0.99 | MSMEG _3763 | 2.63 |
|  | BCG_1725c (Rv1687c) | nr | MSMEG _3762 | 2.04 |
|  | BCG_1786 (Rv1747) | 0.80 | MSMEG_1642 | 0.58 |
|  | BCG_1854c (Rv1819c) | 0.85 | MSMEG_4380 | 1.35 |
| *modA* | BCG_1893 (Rv1857) | 0.73 | MSMEG _2016 | 0.29 |
| *modB* | BCG_1894 (Rv1858) | 0.95 | MSMEG _2015 | 0.10 |
| *modC* | BCG_1895 (Rv1859) | 0.95 | MSMEG _2014 | 0.28 |
| *modD* | BCG_1896 (Rv1860) | 0.84 | MSMEG _3618 | 0.27 |
|  | BCG_2057c (Rv2038c) | nr | MSMEG_5571 | 1.57 |
|  | BCG_2058c (Rv2039c) | 1.18 | MSMEG_0517 | 1.35 |
|  | BCG_2059c (Rv2040c) | 1.14 | MSMEG_0516 | 1.09 |
| *uspA* | BCG_2337 (Rv2316) | 1.31 | MSMEG _4466 | 1.04 |
| *uspB* | BCG_2338 (Rv2317) | 1.22 | MSMEG _4467 | 2.29 |
| *uspC* | BCG_2339 (Rv2318) | 1.28 | MSMEG _4468 | 1.02 |
| *cysA1* | BCG_2412c (Rv2397c) | 1.01 | MSMEG _4530 | 0.23 |
| *cysW* | BCG_2413c (Rv2398c) | 1.24 | MSMEG _4531 | 0.33 |
| *cysT* | BCG_2414c (Rv2399c) | 0.84 | MSMEG _4532 | 0.24 |
| *subI* | BCG_2415c (Rv2400c) | 0.98 | MSMEG _4533 | 0.28 |
|  | BCG_2497c (Rv2477c) | 0.76 | MSMEG_4700 | 0.72 |
|  | BCG_2699c (Rv2686c) | 1.25 | MSMEG _1504 | 4.00 |
|  | BCG_2700c (Rv2687c) | 1.25 | MSMEG _1503 | 3.80 |
|  | BCG_2701c (Rv2688c) | 1.03 | MSMEG _1502 | 5.14 |
| *ugpB* | BCG_2853c (Rv2833c) | 0.85 | MSMEG_5574 | 1.94 |
| *drrA* | BCG_2958 (Rv2936) | 0.95 | MSMEG_6509 | 0.92 |
| *drrC* | BCG_2960 (Rv2938) | 1.10 | MSMEG_6510 | 0.91 |
|  | BCG_3065c (Rv3041c) | 1.10 | MSMEG _2326 | 0.51 |
| *ftsX* | BCG_3126c (Rv3101c) | 0.92 | MSMEG _2090 | 0.47 |
| *ftsE* | BCG_3127c (Rv3102c) | 0.85 | MSMEG _2089 | 0.54 |
| *dppD* | BCG_3721c (Rv3663c) | 0.80 | MSMEG_4357 | 0.72 |
| *dppB* | BCG_3723c (Rv3665c) | 1.22 | MSMEG_0641 | 0.68 |
| *dppA* | BCG_3724c (Rv3666c) | 1.02 | MSMEG_4545 | 1.29 |
| *proZ* | BCG_3815c (Rv3756c) | 1.69 | MSMEG _6331 | 2.06 |
| *proW* | BCG_3816c (Rv3757c) | 1.29 | MSMEG _6332 | 0.88 |
| *proV* | BCG_3817c (Rv3758c) | 1.23 | MSMEG _6333 | 1.48 |
| *proX* | BCG_3818c (Rv3759c) | 1.18 | MSMEG _6334 | 1.07 |
| *rfbE* | BCG_3843 (Rv3781) | 0.95 | MSMEG _6366 | 0.61 |
| *glf*T1 | BCG_3844 (Rv3782) | 1.05 | MSMEG_6367 | 0.38 |
| *rfb*D | BCG_3845 (Rv3783) | 0.83 | MSMEG _6369 | 0.34 |
|  |  |  |  |  |

The corresponding loci in *M. tuberculosis* H37Rv, according to [11], are indicated between brackets in BCG-Pasteur .

| **Table S6 (cont).** Effects of growth rate on the expression of ABC transporters of BCG-Pasteur. | | |
| --- | --- | --- |
| (b) Genes without orthologues found in M. smeg | | |
| **Gene** | **Locus tag** | *r*-value |
|  |  |  |
|  | BCG_0103 (Rv0072) | 0.68 |
|  | BCG_0104 (Rv0073) | 1.17 |
| *ansP2* | BCG_0385c (Rv0346c) | 0.6 |
| *pstS2* | BCG_0985c (Rv0932c) | 0.71 |
|  | BCG_1042 (Rv0987) | 0.90 |
| *cydC* | BCG_1658c (Rv1620c) | 1.36 |
|  | BCG_1778c (Rv1739c) | 1.12 |
|  | BCG_2060c (Rv2041c) | 1.21 |
| *pitB* | BCG_2296 (Rv2281) | 1.02 |
|  | BCG_2585 (Rv2563) | 0.64 |
| *glnQ* | BCG_2586 (Rv2564) | 0.89 |
| *ugpC* | BCG_2852c (Rv2832c) | 1.14 |
| *ugpE* | BCG_2854c (Rv2834c) | 1.29 |
| *ugpA* | BCG_2855c (Rv2835c) | 1.09 |
| *drrB* | BCG_2959 (Rv2937) | 0.93 |
| *dppC* | BCG_3722c (Rv3664c) | 1.15 |
|  |  |  |

The corresponding loci in *M. tuberculosis* H37Rv, according to [11], are indicated between brackets.
