# Supplementary material for: Adaptation of Mycobacteria to Growth Conditions: A Theoretical Analysis of Changes in Gene Expression Revealed by Microarrays
Source: PLoS One. 2013 Apr 12;8(4):e59883. doi: 10.1371/journal.pone.0059883 (PMC3625197; doi:10.1371/journal.pone.0059883)
Supplement: Table S7 — Effects of growth rate on the expression of genes of Msmeg encoding components of ABC transporters without orthologues found in BCG-Pasteur. (DOC) [file pone.0059883.s009.doc]

| **Table S7.** Effects of growth rate on the expression of genes of Msmeg encoding components of ABC transporters without orthologues found in BCG-Pasteur. | | | | | |
| --- | --- | --- | --- | --- | --- |
| Gene | Locus tag | *r*-value | Gene | Locus tag | *r*-value |
|  |  |  | |  |  |
|  | MSMEG_0017 | 0.75 |  | MSMEG_1159 | 0.99 |
|  | MSMEG_0018 | 0.84 |  | MSMEG_1160 | 1.14 |
|  | MSMEG_0133 | 1.06 |  | MSMEG_1216 | 2.19 |
|  | MSMEG_0176 | 0.84 |  | MSMEG_1217 | 1.31 |
|  | MSMEG_0177 | 1.08 |  | MSMEG_1218 | 1.43 |
|  | MSMEG_0211 | 1.54 |  | MSMEG_1219 | 1.19 |
|  | MSMEG_0486 | 1.18 |  | MSMEG_1220 | 1.89 |
|  | MSMEG_0487 | 1.93 |  | MSMEG_1232 | 1.05 |
|  | MSMEG_0488 | 1.18 |  | MSMEG_1233 | 1.07 |
|  | MSMEG_0505 | 1.10 |  | MSMEG_1234 | 0.94 |
|  | MSMEG_0506 | 1.01 |  | MSMEG_1235 | 2.73 |
|  | MSMEG_0507 | 0.99 |  | MSMEG_1372 | 3.93 |
|  | MSMEG_0518 | 1.17 |  | MSMEG_1373 | 3.68 |
|  | MSMEG_0549 | 0.69 |  | MSMEG_1374 | 3.36 |
|  | MSMEG_0551 | 0.73 |  | MSMEG_1613 | 1.22 |
|  | MSMEG_0553 | 0.80 |  | MSMEG_1704 | 1.07 |
|  | MSMEG_0554 | 0.95 |  | MSMEG_1709 | 1.03 |
|  | MSMEG_0555 | 1.14 |  | MSMEG_1711 | 0.80 |
|  | MSMEG_0556 | 1.22 |  | MSMEG_1712 | 0.99 |
|  | MSMEG_0642 | 1.05 |  | MSMEG_2099 | 0.77 |
|  | MSMEG_0646 | 1.10 |  | MSMEG_2499 | 1.06 |
| *phnC* | MSMEG_0647 | 1.07 |  | MSMEG_2501 | 1.31 |
|  | MSMEG_0650 | 1.32 |  | MSMEG_2522 | 1.13 |
|  | MSMEG_0651 | 2.04 |  | MSMEG_2523 | 0.27 |
|  | MSMEG_0658 | 1.22 | *cbiQ* | MSMEG_2609 | 0.89 |
|  | MSMEG_0659 | 2.15 |  | MSMEG_2633 | 3.54 |
|  | MSMEG_0795 | 0.80 |  | MSMEG_2635 | 4.11 |
|  | MSMEG_0796 | 0.96 |  | MSMEG_2844 | 1.34 |
|  | MSMEG_1082 | 0.17 |  | MSMEG_2845 | 1.24 |
|  | MSMEG_1084 | 0.98 |  | MSMEG_2846 | 0.94 |
|  | MSMEG_1085 | 1.10 |  | MSMEG_2927 | 1.37 |
|  | MSMEG_1086 | 1.01 |  | MSMEG_2978 | 0.18 |
|  | MSMEG_1141 | 1.39 |  | MSMEG_2979 | 0.17 |
|  | MSMEG_1142 | 1.05 |  | MSMEG_2981 | 0.12 |

|  | MSMEG_3056 | 0.58 |  | MSMEG_4541 | 1.04 |
| --- | --- | --- | --- | --- | --- |
|  | MSMEG_3057 | 0.35 |  | MSMEG_4543 | 1.14 |
|  | MSMEG_3108 | 0.86 |  | MSMEG_4557 | 0.27 |
| *phrA* | MSMEG_3215 | 0.84 |  | MSMEG_4559 | 0.33 |
|  | MSMEG_3235 | 0.57 |  | MSMEG_4561 | 0.35 |
|  | MSMEG_3236 | 0.67 |  | MSMEG_4586 | 1.46 |
|  | MSMEG_3247 | 0.43 |  | MSMEG_4588 | 1.43 |
|  | MSMEG_3248 | 0.25 |  | MSMEG_4656 | 3.61 |
|  | MSMEG_3249 | 0.24 |  | MSMEG_4657 | 4.53 |
|  | MSMEG_3250 | 0.34 |  | MSMEG_4658 | 3.49 |
|  | MSMEG_3251 | 0.31 |  | MSMEG_4702 | 0.67 |
|  | MSMEG_3268 | 1.17 |  | MSMEG_4761 | 0.66 |
|  | MSMEG_3269 | 1.19 |  | MSMEG_4762 | 0.60 |
|  | MSMEG_3270 | 1.27 |  | MSMEG_4763 | 0.65 |
|  | MSMEG_3277 | 0.47 |  | MSMEG_4764 | 2.55 |
|  | MSMEG_3279 | 0.52 |  | MSMEG_4795 | 1.10 |
|  | MSMEG_3281 | 0.76 |  | MSMEG_5054 | 0.55 |
| *ehuB* | MSMEG_3547 | 1.33 |  | MSMEG_5102 | 1.17 |
| *ehuC* | MSMEG_3548 | 1.41 |  | MSMEG_5147 | 0.74 |
| *ehuD* | MSMEG_3549 | 1.50 |  | MSMEG_5163 | 1.08 |
| *ehuA* | MSMEG_3550 | 1.52 |  | MSMEG_5312 | 0.56 |
|  | MSMEG_3601 | 1.33 |  | MSMEG_5316 | 1.18 |
|  | MSMEG_3636 | 0.92 |  | MSMEG_5318 | 1.19 |
|  | MSMEG_3655 | 0.80 | *ehuB* | MSMEG_5368 | 2.44 |
|  | MSMEG_3656 | 0.64 | *ehuC* | MSMEG_5369 | 1.28 |
|  | MSMEG_3768 | 0.68 | *ehuD* | MSMEG_5370 | 1.02 |
|  | MSMEG_3999 | 1.41 | *ehuA* | MSMEG_5371 | 1.24 |
|  | MSMEG_4000 | 0.88 |  | MSMEG_5531 | 1.00 |
|  | MSMEG_4098 | 1.15 |  | MSMEG_5572 | 1.60 |
|  | MSMEG_4099 | 1.06 |  | MSMEG_5573 | 1.75 |
|  | MSMEG_4100 | 0.68 |  | MSMEG_5660 | 0.87 |
|  | MSMEG_4101 | 0.93 |  | MSMEG_5661 | 0.83 |
|  | MSMEG_4354 | 1.03 |  | MSMEG_6018 | 2.92 |
|  | MSMEG_4355 | 0.82 |  | MSMEG_6019 | 2.50 |
|  | MSMEG_4356 | 0.97 |  | MSMEG_6020 | 2.43 |
|  | MSMEG_4385 | 1.05 |  | MSMEG_6045 | 1.13 |
|  | MSMEG_4386 | 1.20 |  | MSMEG_6046 | 1.11 |
|  | MSMEG_4387 | 0.98 |  | MSMEG_6047 | 0.89 |
|  | MSMEG_4388 | 1.35 |  | MSMEG_6052 | 0.94 |
|  | MSMEG_4470 | 0.91 |  | MSMEG_6122 | 3.29 |

|  | MSMEG_6123 | 2.73 |  | MSMEG_6724 | 0.40 |
| --- | --- | --- | --- | --- | --- |
|  | MSMEG_6124 | 2.74 |  | MSMEG_6725 | 0.54 |
|  | MSMEG_6309 | 0.70 |  | MSMEG_6726 | 0.60 |
|  | MSMEG_6494 | 1.44 |  | MSMEG_6748 | 0.78 |
|  | MSMEG_6495 | 1.39 |  | MSMEG_6765 | 1.18 |
|  | MSMEG_6496 | 1.48 |  | MSMEG_6766 | 0.69 |
|  | MSMEG_6522 | 0.54 |  | MSMEG_6802 | 1.33 |
|  | MSMEG_6523 | 0.64 |  | MSMEG_6804 | 1.95 |
|  | MSMEG_6524 | 0.85 |  | MSMEG_6865 | 1.71 |
|  | MSMEG_6594 | 0.83 |  | MSMEG_6867 | 1.53 |
|  | MSMEG_6668 | 1.18 |  | MSMEG_6880 | 0.15 |
|  | MSMEG_6669 | 1.22 |  | MSMEG_6909 | 1.15 |
|  | MSMEG_6670 | 1.05 |  | MSMEG_6911 | 1.13 |
|  |  |  | |  |  |
