# Supplementary material for: Adaptation of Mycobacteria to Growth Conditions: A Theoretical Analysis of Changes in Gene Expression Revealed by Microarrays
Source: PLoS One. 2013 Apr 12;8(4):e59883. doi: 10.1371/journal.pone.0059883 (PMC3625197; doi:10.1371/journal.pone.0059883)
Supplement: Table S8 — Effects of growth rate on the expression of genes of serine/threonine protein kinases. (DOC) [file pone.0059883.s010.doc]

| **Table S8.** Effects of growth rate on the expression of genes of serine/threonine protein kinases. | | | | | |
| --- | --- | --- | --- | --- | --- |
| BCG-Pasteur | | | Msmeg | | |
| Gene | Locus tag | *r*-value | Gene | Locus tag | *r*-value |
|  |  |  |  |  |  |
| *pkn*A | BCG_0045c (Rv0015c)* | nr | *pkn*A | MSMEG_0030* | 0.69 |
| *pkn*B | BCG_0044c (Rv0014c)* | 0.83 | *pkn*B | MSMEG_0028* | 0.85 |
|  |  |  | *pkn*B | MSMEG_5437 | 0.93 |
| *pkn*D | BCG_0983c (Rv0931c) | 0.67 |  |  |  |
|  |  |  | *pkn*D | MSMEG_1190 | 1.67 |
|  |  |  | *pkn*D | MSMEG_1200 | 1.48 |
|  |  |  | *pkn*D | MSMEG_0886 | 2.08 |
| *pkn*E | BCG_1782 (Rv1743) | 0.79 |  |  |  |
|  |  |  | *pkn*E | MSMEG_1215 | 1.10 |
|  |  |  | *pkn*E | MSMEG_5513 | 0.65 |
| *pkn*F | BCG_1785 (Rv1746)* | 0.90 |  | MSMEG_3677* | 1.31 |
| *pkn*G | BCG_0449c (Rv0410c)* | 1.05 | *pkn*G | MSMEG_0786* | 1.91 |
|  |  |  | *pkn*H | MSMEG_4554 | 2.07 |
|  |  |  | *pkn*H | MSMEG_4595 | 0.71 |
| *pkn*H | BCG_1325c (Rv1266c)* | 0.88 | *pkn*D | MSMEG_4366* | 0.93 |
| *pkn*I | BCG_2935c (Rv2914c) | 1.23 |  |  |  |
| *pkn*J | BCG_2108 (Rv2088) | 1.32 |  |  |  |
| *pkn*K | BCG_3105c (Rv3080c)* | 1.51 | *pkn*K | MSMEG_0529* | 4.34 |
| *pkn*L | BCG_2191 (Rv2176)* | 0.91 | *pkn*L | MSMEG_4243* | 1.11 |
|  |  |  |  |  |  |

nr, no result; *, orthologues found in BCG-Pasteur and Msmeg are located in the same line.

The corresponding loci in *M. tuberculosis* H37Rv, according to [11], are indicated between brackets in BCG-Pasteur .
